# Rational construction of interfacial photocatalysts toward outstanding performance in energy and evironmental applications

**DOI:** 10.1016/j.isci.2026.117358

**Published:** 2026-09-04

**Authors:** Chengjie Chen, Qian Wang, Hongbo Cui, Guijian Guan, Ming-Yong Han

**Affiliations:** 1 Institute of Molecular Plus, Tianjin University, Tianjin 300072, P.R. China; 2 Key Laboratory of Advanced Energy Materials Chemistry (Ministry of Education), Nankai University, Tianjin 300071, P.R. China

**Keywords:** photocatalysis, interfacial engineering, charge transfer dynamics, solar-to-fuel conversion, waste upcycling

## Abstract

Interfacial photocatalysts with precisely engineered junctions are critical for overcoming the performance limitations of conventional photocatalytic systems, as they enable efficient charge transfer, suppress carrier recombination, and enhance surface redox kinetics. This review first outlines the rational design principles for interfacial photocatalysts and precise interfacial control, highlighting advanced engineering strategies—including asymmetric single-atom doping, construction of a step-scheme built-in electric field, and spatial decoupling of dual cocatalysts. We then decipher the underlying physicochemical mechanisms using *operando* characterizations and theoretical simulations, transforming the traditional “black-box” catalysis into a visualized six-step cascade of charge transfer and molecular activation. The transformative utility of these interfaces is demonstrated in high-efficiency energy conversion and environmental remediation, with a particular focus on the paradigm shift from passive mineralization of emerging contaminants to proactive “Waste-to-Wealth” upcycling of plastics and biomass. Finally, we discuss challenges and future directions in reactor scale-up, durability, and AI-driven materials discovery.

## Graphical abstract

**Figure:**
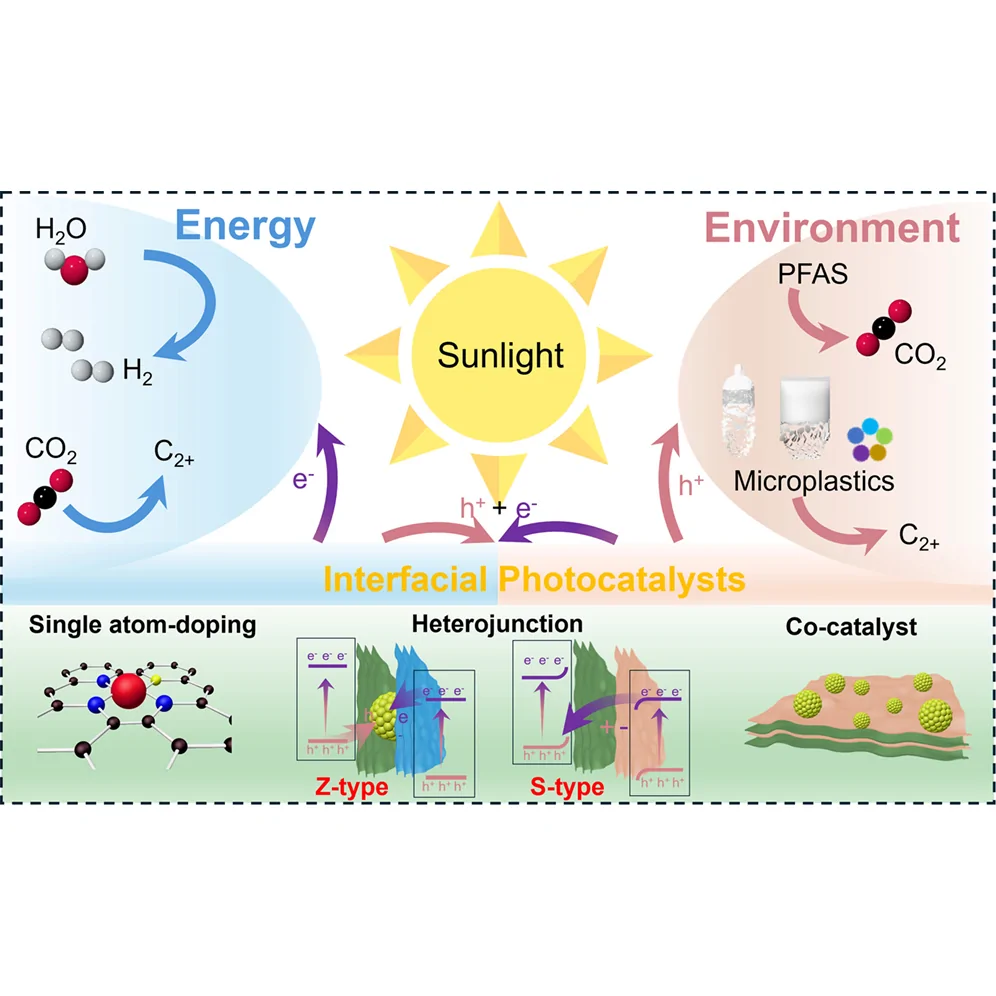

## Teaser

Catalysis; Electrochemical energy conversion; Environmental science; Materials science

## Introduction

Since the landmark demonstration of photocatalytic water splitting on TiO_2_ electrodes by Fujishima and Honda, photocatalysis has evolved from fundamental mechanistic inquiries into a multifaceted discipline aimed at utilizing solar energy to address energy and environmental problems.^1^ Through artificial photosynthesis, photocatalysts can drive the reductive conversion of greenhouse gases into value-added fuels (e.g., CH_4_, C_2_H_4_), or trigger water splitting for clean hydrogen production.^2^ Concurrently, the robust reactive oxygen species (ROS) and charge carriers generated at the photocatalyst surface offer a potent pathway not only for the deep mineralization of recalcitrant emerging contaminants (e.g., fluorinated compounds and plasticizers) but also for the high-value upcycling of waste plastics.^3^^,^^4^^,^^5^ Currently, the escalating global imperatives to achieve a carbon-neutral energy economy and mitigate severe ecological degradation have positioned photocatalysis at the vanguard of contemporary materials science and chemical engineering.^6^^,^^7^^,^^8^^,^^9^

To efficiently convert solar energy, semiconductors serve as the indispensable core units of photocatalytic reactions. However, practical photocatalytic technology remains constrained by a persistent “efficiency ceiling”, which arises from a cascade of interdependent processes spanning femtosecond to millisecond timescales. Traditional semiconductors (e.g., pristine TiO_2_,^10^ CdS;^11^ and graphitic carbon nitride (g-C_3_N_4_)^12^) are universally plagued by three intrinsic bottlenecks. First, optical harvesting deficits: most stable semiconductors possess wide bandgaps, severely restricting their photo-absorption to the ultraviolet (UV) region and wasting the majority of the solar spectrum.^13^ Second, sluggish carrier dynamics: driven by strong Coulombic interactions, photogenerated electron-hole pairs often undergo ultrafast non-radiative recombination within the bulk, drastically shortening their diffusion lengths.^14^ Third, kinetic impedance at surface sites: multi-electron proton-coupled electron transfer (PCET) reactions (such as the 8-electron reduction of CO_2_ to CH_4_) encounter formidable activation energy barriers, where the sluggish kinetics of surface intermediates fail to match the rapid carrier generation rate.^15^^,^^16^

To shatter these bottlenecks, the research paradigm has decisively pivoted from the empirical exploration of bulk photocatalysts to the precise, atomic-level manipulation of interfacial microenvironments.^17^^,^^18^^,^^19^ The term “interfacial photocatalyst” denotes a composite photocatalytic system featuring precisely engineered junctions between two or more distinct components—including semiconductors, cocatalysts, conductive scaffolds, or molecular entities—where the interface functions as the dynamic hub for photogenerated charge separation, reactant adsorption and activation, and product desorption, rather than serving merely as a passive physical boundary. This review encompasses three major categories of interfacial systems: (1) doping-modulated micro-interfaces within a single semiconductor matrix, (2) heterojunction macro-interfaces formed between two or more semiconductors (with particular emphasis on S-scheme architectures), and (3) cocatalyst/semiconductor interfaces designed for spatially decoupled redox reactions. In advanced interfacial photocatalysts, it functions as the dynamic hub where photogenerated charges are spatially separated, reactants are activated at lowered energy barriers, and products are efficiently desorbed. This unique multifunctionality endows interfacial photocatalysts with decisive advantages over conventional bulk materials, including prolonged carrier lifetimes, enhanced surface redox kinetics, and superior resistance to photocorrosion.^20^^,^^21^^,^^22^ More importantly, the modern era of interfacial photocatalysis is defined by the transition from static structural design to dynamic *operando* tracking. The deployment of ultrafast spectroscopy and *in situ* characterization techniques has fundamentally demystified the catalytic pathways, providing fingerprint-level evidence of transient charge flow and molecular activation.^23^^,^^24^ By focusing on the unifying role of the interface in directing charge flow and catalytic turnover, we aim to provide a coherent framework that bridges atomic-scale electronic modulation with macroscopic photocatalytic performance.

While several excellent reviews have covered photocatalytic heterojunctions, S-scheme charge transfer, single-atom engineering, or cocatalyst design, the present review is organized around three integrated perspectives. First, we provide a unified framework that traces the complete interfacial design chain—from atomic-level doping and built-in electric field construction to spatially decoupled dual cocatalysts—and systematically connect these design principles with the full six-step photocatalytic cycle spanning from photon harvesting to product desorption. Second, we place a unique emphasis on the paradigm shift from static structural design to dynamic *operando* mechanistic verification, critically summarizing the “gold standard” of *in situ* irradiated X-ray photoelectron spectroscopy, femtosecond transient absorption spectroscopy, and *in situ* Fourier transform infrared spectroscopy as indispensable tools for validating interfacial charge-transfer pathways. Third, we highlight a timely and rarely reviewed transition in environmental photocatalysis: from the conventional passive mineralization of pollutants to the proactive “Waste-to-Wealth” upcycling of plastics and biomass, which represents a conceptually distinct and application-driven frontier. By bridging precise atomic synthesis, *operando* kinetic tracking, and macroscopic chemical engineering, a comprehensive roadmap is delineated for developing the next-generation photocatalytic systems.

## Interface construction and functionality

### Limitations of traditional semiconductors as a prelude to interfacial photocatalysts

**Figure 1:**
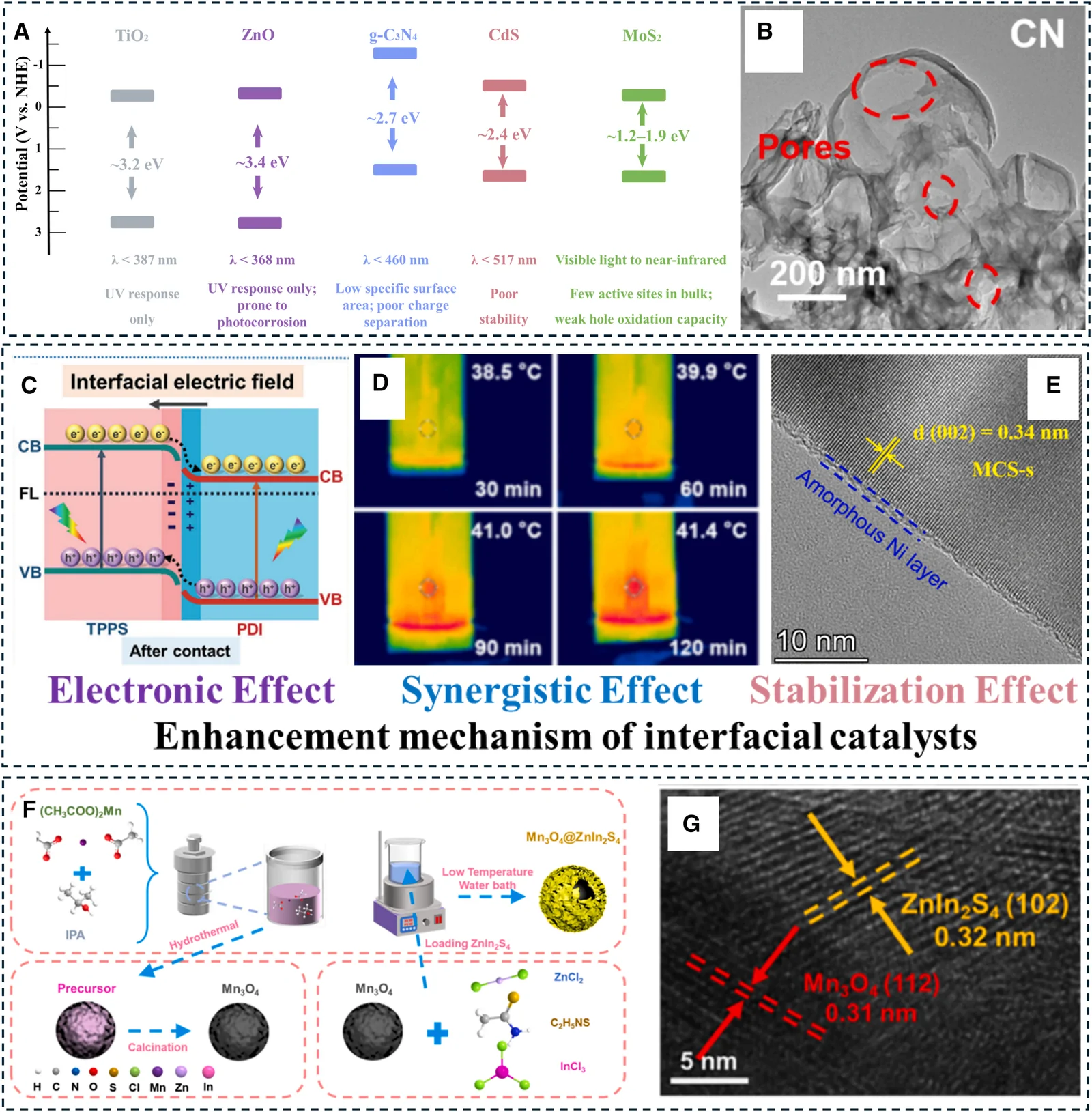
Fundamentals and enhancement mechanisms of interfacial photocatalysts (A) Band structures, light-absorption profiles, and charge-separation dynamics of traditional single-component photocatalysts. (B) Transmission electron microscopy (TEM) image of CN (Scale bar represents 200 nm).^30^ Copyright 2026 Elsevier Ltd. Schematic diagrams depicting interfacial enhancement mechanisms via the (C) electronic effect^31^; Copyright 2022 Wiley-VCH GmbH. (D) synergistic effect^30^ and (E) interfacial stabilization effect (Scale bar represents 10 nm).^32^ Copyright 2022 Elsevier Ltd. Schematic illustration (F) and high-resolution TEM (HRTEM) image (G) of the Mn_3_O_4_@ZnIn_2_S_4_ core-shell heterojunction (Scale bar represents 5 nm).^33^ Copyright 2024 Elsevier Ltd.

Traditional semiconductors, primarily metal oxides and sulfides, have long served as the foundational pillars of photocatalytic research due to their well-established synthesis protocols and unambiguous band structures^25^^,^^26^^,^^27^^,^^28^^,^^29^ Figure 1A illustrates the band gap structures and catalytic properties of these traditional materials. As the quintessential model photocatalyst, TiO_2_ exhibits a catalytic performance that is fundamentally dictated by its crystalline phases (predominantly anatase and rutile) and micro/nano morphologies (e.g., nanoparticles, nanorods, and nanotubes). While morphological tuning effectively modulates its surface properties and active site density, TiO_2_ is intrinsically constrained by its wide bandgap of approximately 3.2 eV.^34^ This restricts its optical absorption strictly to the UV spectrum, constituting its most critical performance bottleneck. ZnO, often considered a highly competitive alternative to TiO_2_, boasts superior intrinsic electron mobility, environmental benignity, and low cost. Through morphology engineering and elemental doping, its charge separation dynamics can be significantly improved. However, ZnO shares a similarly wide bandgap (∼3.37 eV) and suffers from severe photocorrosion under continuous illumination, drastically limiting its long-term operational stability.^35^

To harness the visible spectrum, metal sulfides have emerged as highly efficient alternatives due to their intrinsically narrower bandgaps. MoS_2_, a prototypical two-dimensional (2D) transition metal dichalcogenide, features a tunable bandgap (1.2–1.9 eV) that is highly responsive to visible light.^36^ Its catalytic prowess originates primarily from the coordinatively unsaturated sulfur atoms located at its structural edges. Exfoliating bulk MoS_2_ into single- or few-layer nanosheets can drastically amplify its specific surface area and expose a higher density of active edge sites. Nonetheless, its overall efficiency is severely hampered by rapid electron-hole recombination and a fundamental scarcity of active sites, a problem exacerbated by its large, catalytically inert basal plane. Conversely, CdS is a classical visible-light-driven catalyst renowned for its exceptional efficiency in photocatalytic hydrogen evolution. With a bandgap of approximately 2.4 eV, its conduction and valence band edges thermodynamically straddle the water redox potentials. Despite its exceptional initial activity, pristine CdS is plagued by ultrafast charge carrier recombination and catastrophic photocorrosion, wherein photogenerated holes self-oxidize the lattice S^2−^ ions, leading to rapid deactivation. Furthermore, its inherent biological toxicity presents a formidable barrier to widespread environmental application.

In recent years, metal-free carbon-based materials, predominantly g-C_3_N_4_, have garnered massive attention. Composed of Earth-abundant carbon and nitrogen, g-C_3_N_4_ is a highly stable, eco-friendly 2D polymeric semiconductor with a moderate bandgap of ∼2.7 eV, enabling visible-light harvesting.^12^^,^^37^ Structural engineering strategies, such as exfoliating bulk g-C_3_N_4_ into ultrathin nanosheets or constructing hierarchical porous frameworks, have proven effective in maximizing the specific surface area and increasing the density of exposed active sites. For example, as shown in Figure 1B, graphene-based semiconductor architectures possess an ultrathin, porous nanosheet structure, which facilitates the exposure of a greater number of active edge sites.^30^ Despite these advantages, the practical utility of g-C_3_N_4_ remains severely restricted by its moderate visible-light utilization efficiency (often accompanied by absorption edge blue-shifts in ultrathin forms), an inherently low specific surface area in its bulk state, and severe intra- and inter-planar carrier recombination.

In summary, the catalytic efficacy of traditional monolithic photocatalysts is universally constrained by three intrinsic bottlenecks: narrow optical response ranges, rapid charge carrier recombination, and poor photochemical stability (e.g., photocorrosion). These inherent deficiencies have catalyzed an inevitable paradigm shift in the field, driving the transition from single-component systems to advanced composite architectures. By integrating disparate materials through interface engineering, researchers can harness synergistic effects to bypass these thermodynamic and kinetic limitations. Specifically, interfacial composite architectures leverage three distinct regulatory mechanisms (Figures 1C–1E):

First, electronic structure modulation tailors the energy band alignment and constructs a robust built-in electric field, thereby broadening the light-harvesting spectrum and expediting charge transfer dynamics. For instance, Zhu and co-workers (Figure 1C) successfully constructed a stable electron donor-acceptor (D-A) interface. This rationally designed heterojunction exhibited a remarkably enhanced photocatalytic H_2_ evolution rate of 30.36 mmol h^−1^ g^−1^, which is nearly an order of magnitude higher than those of its pristine counterparts. This dramatic performance leap is fundamentally attributed to the generation of a giant internal electric field at the D-A interface—approximately 3-fold stronger than that of the single components. This built-in field profoundly facilitates the spatial separation of charge carriers and significantly prolongs their excited-state lifetimes, providing a paradigm for designing highly active D-A interfacial materials.^31^

Second, multi-field synergistic coupling integrates exogenous stimuli (such as magnetic, electric, or optical fields) to harness localized thermal effects, profoundly amplifying the overall efficiency and utilization of the solar spectrum. As an overarching illustration of this concept (Figure 1D), Li’s group engineered a photothermal-assisted photocatalytic system by anchoring carbon dots (CDs) onto black g-C_3_N_4_ (CN-B-CDs).^30^ Photothermal imaging confirmed that the dual localized heat sources significantly elevate the interfacial temperature. Consequently, the CN-B-CDs system not only achieved highly efficient H_2_ production but also demonstrated exceptional structural stability and adaptability across complex aqueous environments, underscoring its viability for industrial-scale deployment. Further demonstrating this principle, Shi and co-workers engineered a Mn_3_O_4_@ZnIn_2_S_4_ S-scheme heterojunction via a facile wet-chemical approach (Figures 1F and 1G).^33^ Crucially, the application of an external magnetic field triggered a profound synergistic coupling between the magnetothermal effect and the intrinsic photothermal response of Mn_3_O_4_. This dual-stimuli synergy elevated the localized interfacial temperature, thermodynamically driving the spatial separation of photogenerated electron-hole pairs and accelerating sluggish surface kinetics.

Finally, interfacial stabilization engineering—achieved via the construction of core-shell topologies or the strategic integration of protective overlayers—serves to simultaneously preserve structural integrity and sustain long-term catalytic activity. A compelling demonstration is provided by Huang and colleagues (Figure 1E), who engineered a Ni/S-vacancy-rich Mn_0.3_Cd_0.7_S heterostructure to overcome both kinetic and stability limitations in demanding environments.^32^ While metal/semiconductor Schottky barriers typically facilitate charge separation, they can inadvertently impede electron migration across the interface. By strategically coupling plasmonic Ni with S vacancies, the authors effectively lowered the Schottky barrier, enabling the seamless injection of hot electrons. This configuration yielded an outstanding H_2_ production rate of 164.1 mmol h^−1^ g^−1^ in simulated seawater alongside an impressive apparent quantum yield (AQY) of 60.4% at 420 nm. Most crucially, the spatially integrated Ni layer functions as a robust protective shield, effectively thwarting the photocorrosion of the underlying sulfide matrix and ensuring an enduring operational stability under harsh saline conditions.

Overall, this evolution—from isolated semiconductors to intricately designed heterojunctions—establishes a robust and necessary foundation for the development of next-generation, high-performance photocatalytic systems.

### Doping engineering to modulate local interfacial electronic structures

Doping modulation is a strategy involving the introduction of foreign elements into a semiconductor lattice to alter its electronic structure and band configuration.^38^ The band gap and band edge positions of semiconductor photocatalysts determine their light absorption range and redox capabilities; doping serves to effectively strike a balance between these two critical parameters.^39^^,^^40^ The selection of doping elements requires a comprehensive assessment of factors such as ionic radius compatibility, electronegativity differences, doping concentration, and the specific band requirements of the target reaction. From the perspective of charge transfer efficiency, an ideal doping element should be capable of: (1) forming effective impurity levels that facilitate the separation of photogenerated electron-hole pairs; (2) avoiding acting as recombination centers—meaning the impurity levels should not be situated within the central region of the forbidden band; and (3) improving the electron density distribution at surface active sites.^41^^,^^42^^,^^43^ Regarding the modulation of electronic structure, the primary role of doping is manifested through impurity levels acting as “stepping stones” to narrow the effective band gap and thereby extend the visible-light response range.^44^

**Figure 2:**
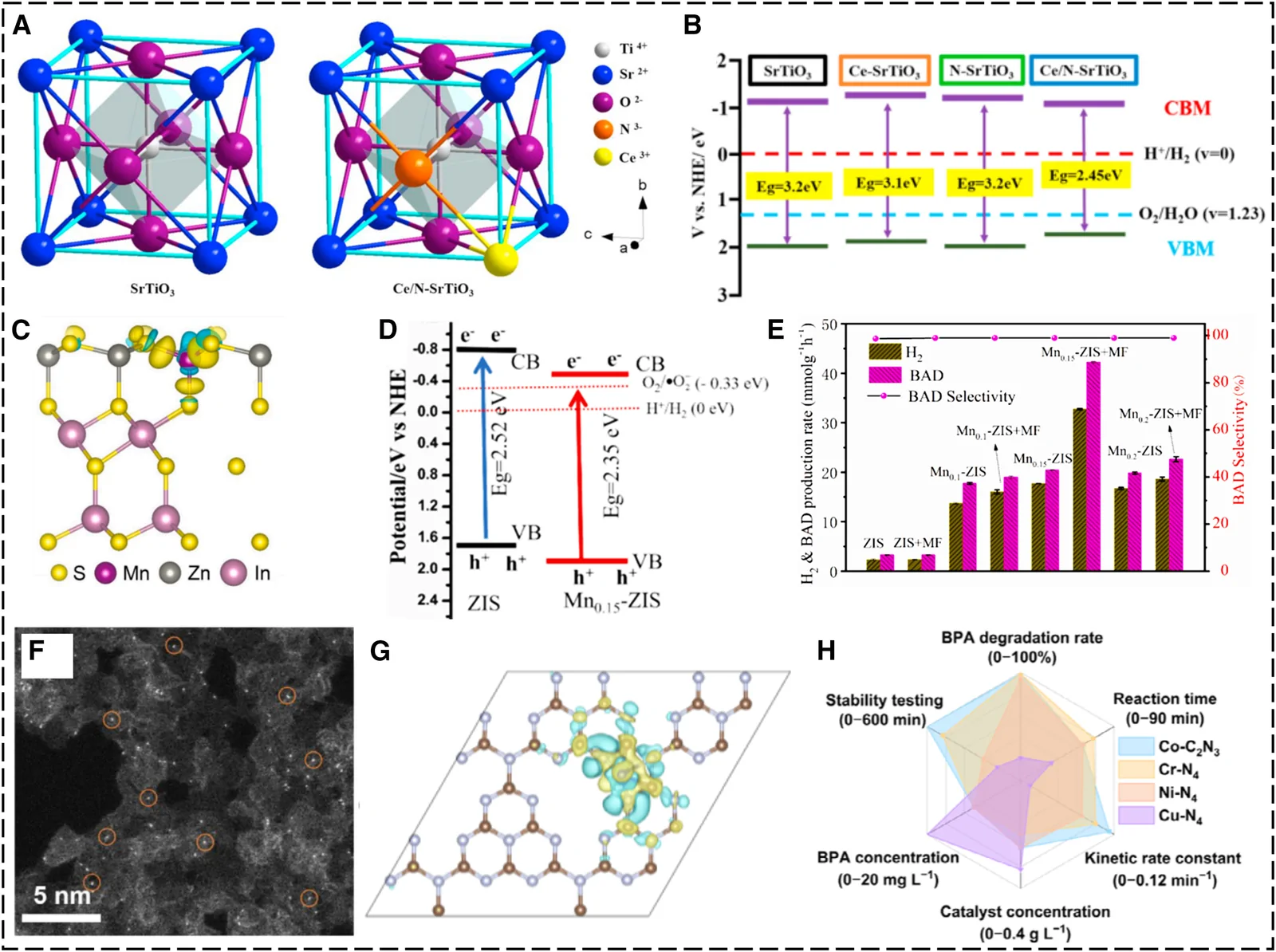
Modulation of local interfacial electronic structures via elemental doping and single-atom coordination (A) Crystal structures of pristine and Ce/N co-doped SrTiO_3_. (B) Energy band alignments of the corresponding SrTiO_3_ samples.^46^ Copyright 2022 Elsevier Ltd. (C) Differential charge density map of Mn_0.15_-ZIS. (D) Schematic illustrating the bandgap modulation induced by Mn dopants. (E) Photocatalytic H_2_ and benzaldehyde (BAD) production rates, alongside BAD selectivity, for various photocatalysts in the presence and absence of a magnetic field.^47^ Copyright 2025 Elsevier Ltd. (F) HAADF-STEM image of Co_SA_-CN (Scale bar represents 5 nm). (G) Charge density difference mapping of the coordination atoms in the asymmetric Co-C_2_N_3_ structure. (H) Comparison of photocatalytic bisphenol A (BPA) degradation performance between the asymmetric Co-C_2_N_3_ catalyst and conventional symmetric M-N_4_ structures.^48^ Copyright 2025 Wiley-VCH GmbH.

Non-metal dopants are predominantly utilized to elevate the valence band maximum (VBM) by hybridizing their p-orbitals with those of the host lattice (e.g., the O 2p orbitals in metal oxides). This upward shift of the VBM significantly narrows the intrinsic bandgap, extending the optical absorption well into the visible and near-infrared (NIR) regions. R. Asahi and his collaborators demonstrated that doping TiO_2_ with N leads to the hybridization of N 2p orbitals with O 2p orbitals; this, in turn, causes an upward shift of the valence band, thereby significantly narrowing the band gap of TiO_2_ and rendering it active under visible light.^45^ As shown in Figures 2A and 2B, Wang and collaborators synthesized Ce/N co-doped SrTiO_3_ via a hydrothermal method and applied it to hydrogen production.^46^ The introduction of Ce and N ions effectively narrowed the band gap of SrTiO_3_, thereby significantly enhancing both the photo response range and the photocurrent. While bulk elemental doping has historically been employed to narrow the wide bandgaps of traditional semiconductors, contemporary research has decisively shifted toward interfacial doping engineering.^49^^,^^50^ At the interface, introducing heteroatoms does not merely alter the macroscopic optical response; rather, it fundamentally reconfigures the local electronic environment, shifts the Fermi level, and creates highly active polarization centers. By strategically incorporating metal or non-metal dopants at the junction of composite materials, researchers can simultaneously overcome thermodynamic barriers for light harvesting and kinetic barriers for charge transfer. More importantly, these electronegative heteroatoms at the interface induce asymmetric charge distribution. Currently, the construction of such interfacial asymmetric sites has demonstrated unique advantages across various catalytic fields.^51^^,^^52^ Fundamentally, the introduction of electronegative atoms at the interface induces localized coordination defects and triggers a redistribution of interfacial charge; consequently, this heterogeneous interface—characterized by coordination unsaturation—exhibits exceptionally high reactivity. For example, as illustrated in Figures 2C–2E, He and collaborators introduced spin-polarized Mn atoms into a ZnIn_2_S_4_ matrix.^47^ This strategic doping induced a redistribution of surface charge and narrowed the bandgap, effectively establishing Mn sites as highly active catalytic centers. These localized Mn sites preferentially accumulated holes to activate benzyl alcohol, while an exceptionally favorable direct H^+^ transfer pathway facilitated synchronous H_2_ generation at adjacent sites, demonstrating superior dual-functional catalytic performance. Furthermore, Tang et al. extended this concept by depositing metallic bismuth (Bi) nanoparticles onto oxygen-doped ZnIn_2_S_4_ nanosheets.^53^ Beyond merely reducing the bandgap, the Bi modification significantly amplified the photocatalytic activity via a pronounced surface plasmon resonance effect, effectively broadening the utilization of the visible-light spectrum.

In recent years, interfacial single-atom catalysis (SAC) has emerged as the most sophisticated frontier in this domain. Unlike the random distribution of aggregated metal clusters, substituting specific lattice sites at the interface with isolated metal atoms maximizes atom utilization (approaching 100%) and provides geometrically well-defined, coordinatively unsaturated active sites.^54^ For instance, Lazaar et al. engineered a g-C_3_N_4_ matrix featuring a remarkably low loading of single-atom Pt via a direct deposition method.^55^ As validated by aberration-corrected electron microscopy and extended X-ray absorption fine structure analyses, the Pt species were atomically dispersed. Comparative performance analyses revealed that this low-loading SAC substantially outperformed its nanoparticle-supported counterpart prepared at a higher loading. Specifically, the optimized single-atom Pt/C_3_N_4_ composite achieved a hydrogen production rate approximately 5.9 times higher than that of the Pt nanoparticle-modified system, underscoring the extraordinary intrinsic activity of isolated atoms. Despite these advancements, it has been increasingly recognized that highly symmetric metal coordination structures can induce kinetic hysteresis during charge transfer. To bypass this limitation, single-atom catalysts featuring asymmetric coordination microenvironments have emerged as a cutting-edge solution. Leveraging a spatial confinement strategy, Liu et al. successfully constructed a single-atom cobalt catalyst (Co-C_2_N_3_) with an asymmetric coordination configuration (Figure 2F).^48^ This unique interfacial microenvironment not only optimizes carrier separation pathways but also engenders electron-rich localized active sites (Figure 2G). Consequently, these sites effectively activate molecular oxygen to generate potent ROS, such as superoxide radicals. Through the synergy of optimized charge kinetics and multi-site interplay, this asymmetric catalyst facilitated a highly efficient two-stage kinetic process for BPA degradation, significantly surpassing the performance of traditional single-stage carbon nitride systems (Figure 2H). Ultimately, these single-atom dopants induce intense localized internal electric fields driven by electronegativity differentials between the dopant and the host matrix. These polarized metallic centers function as highly efficient interfacial “electron pumps”, directionally extracting photogenerated electrons from the bulk phase to the reactive surface, thereby drastically suppressing bulk recombination. In essence, interfacial doping transforms a passive physical boundary into an active, electronically asymmetric reaction hub. By meticulously selecting the type, concentration, and spatial distribution of dopants, researchers can establish a bespoke electronic microenvironment that serves as the foundation for the more complex heterojunction architectures discussed in the following section.

### Heterojunction interface to generate built-in electric fields for spatial charge separation

While doping optimizes the local electronic environment at the atomic scale, addressing the macroscopic spatial separation of photogenerated carriers necessitates the construction of sophisticated heterojunction interfaces.^56^ When two semiconductors with matching band structures are coupled, the difference in their work functions induces spontaneous electron diffusion across the interface until Fermi level equilibration is achieved. This interfacial charge redistribution triggers profound band bending and establishes a robust built-in electric field (IEF), which serves as the fundamental thermodynamic driving force for directional charge transport.^57^

**Figure 3:**
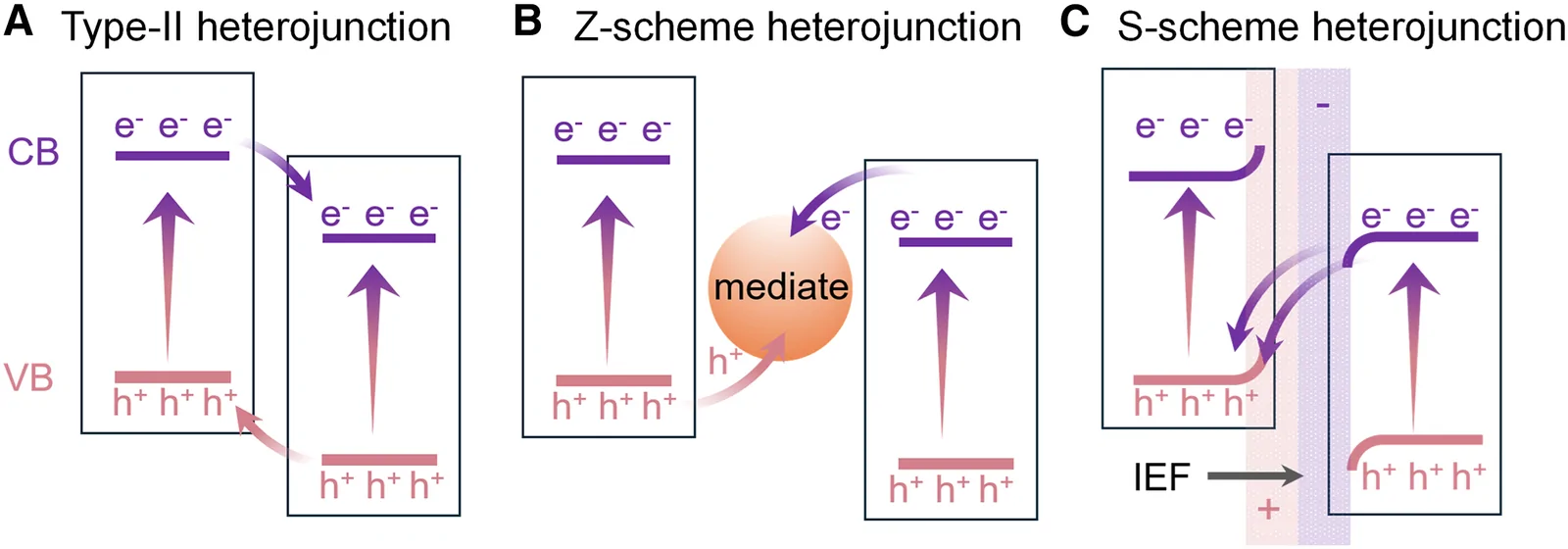
Interfacial heterojunction architectures and their charge transfer mechanisms Schematic illustrations of energy band alignments and photogenerated charge migration pathways in (A) conventional Type-II, (B) Z-scheme, and (C) S-scheme heterojunctions.

Historically, the design of heterojunctions evolved from the conventional Type-II architecture (Figure 3A). In a typical Type-II system, staggered band alignment drives photogenerated electrons to the semiconductor with the more positive conduction band (CB) and holes to the one with the more negative valence band (VB).^58^^,^^59^^,^^60^ Although this achieves the spatial isolation of charge carriers, it suffers from a fundamental thermodynamic trade-off: electrons and holes inevitably accumulate at the lowest available energy levels, thereby severely sacrificing the overall redox capability of the system.

To bypass this thermodynamic dilemma, the research paradigm has decisively shifted toward the Z-scheme architecture (Figure 3B) and, more recently, S-scheme heterojunctions (Figure 3C). Conceptually inspired by natural photosynthesis, a classical Z-scheme photocatalytic system consists of two distinct semiconductors with staggered band structures, connected via an electron-transfer mediator.^61^^,^^62^ Upon photoexcitation, photogenerated electrons from the semiconductor with the lower CB migrate across the conductive mediator to rapidly recombine with the photogenerated holes in the semiconductor with the higher VB.^63^^,^^64^^,^^65^ This vectorial charge transfer mechanism elegantly achieves efficient spatial separation of electron-hole pairs while strictly preserving the electrons with the highest reduction potential and the holes with the highest oxidation potential.

**Figure 4:**
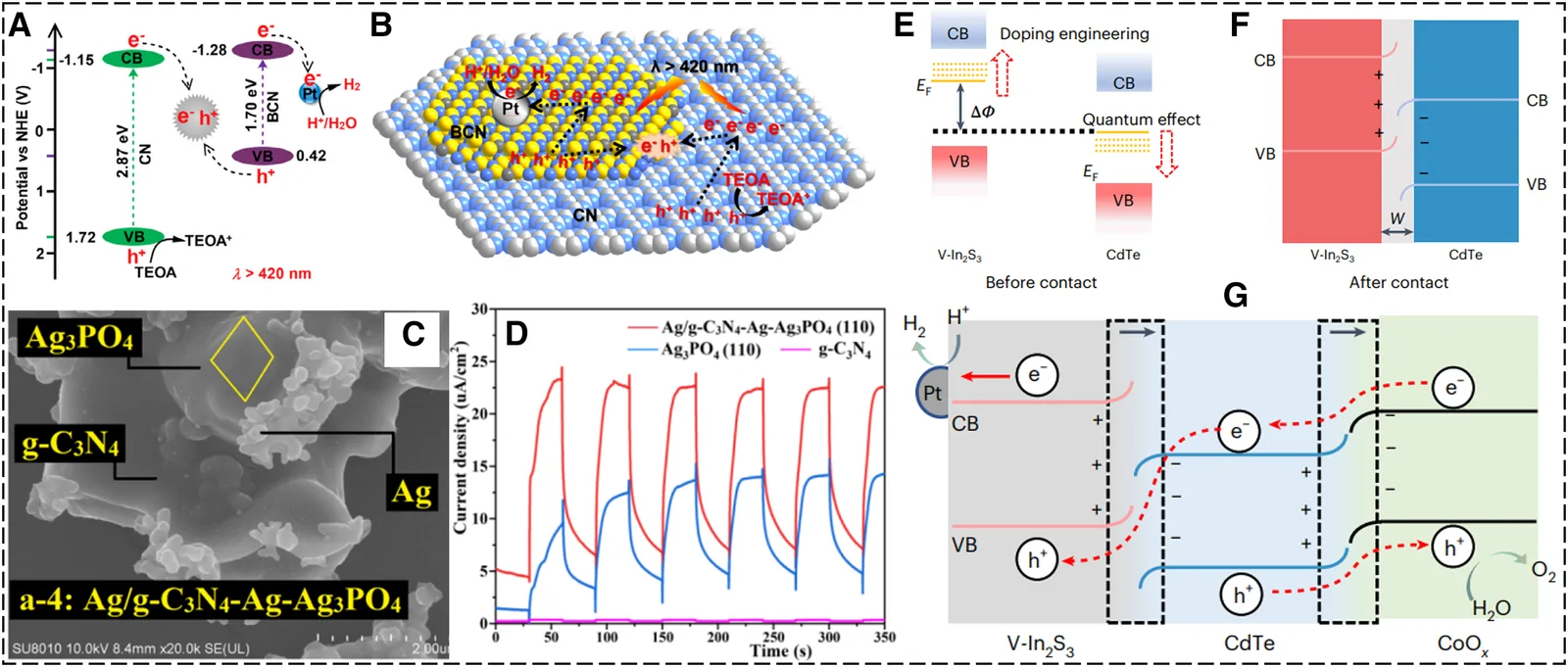
Design and charge transfer mechanisms of advanced Z-scheme heterojunctions (A) Schematic of the energy band alignment and photogenerated charge migration pathway in a Z-scheme heterojunction. (B) Photocatalytic H_2_ evolution mechanism over the 2D/2D CN/BCN van der Waals (vdW) heterojunction.^66^ Copyright 2020 Elsevier Ltd. Characterization and performance of the Ag/g-C_3_N_4_-Ag-Ag_3_PO_4_ (110) composite: (C) scanning electron microscopy (SEM) image (Scale bar represents 2 μm), and (D) transient photocurrent.^67^ Copyright 2020 Elsevier Ltd. Energy band structures of CdTe and V-In_2_S_3_ (E) before and (F) after contact. (G) Schematic of photoinduced carrier transfer and the overall water splitting process over the CdTe/V-In_2_S_3_ hybrid.^68^ Copyright 2023 Springer Nature Ltd.

Recent advancements have produced highly efficient Z-scheme systems utilizing this principle (Figure 4A). For instance, Dong et al. constructed a metal-free 2D/2D carbon nitride/carbon-doped boron nitride (CN/BCN) Z-scheme heterojunction, utilizing interfacial Pt species to facilitate charge bridging (Figure 4B).^66^ This precise interfacial engineering significantly accelerated the directed transport and spatial separation of charge carriers. As a result of this optimized kinetic pathway, the resulting composite delivered an outstanding photocatalytic hydrogen evolution rate of 3357.1 μmol h^−1^ g^−1^. Similarly, Li et al. engineered an all-solid-state Ag/g-C_3_N_4_-Ag-Ag_3_PO_4_ photocatalyst via a photo-assisted isoelectric point strategy (Figure 4C). Benefiting from the robust Z-scheme charge transfer mediated by interfacial Ag nanoparticles, the catalyst exhibited an enhanced photocurrent response, broadened visible-light harvesting, and exceptional charge separation efficiency (Figure 4D). Consequently, the system demonstrated potent redox capabilities, maintaining a stable degradation rate of over 80% for the photocatalytic mineralization of organic pollutants.^67^

Beyond traditional architectures, advanced conjugated and defect-engineered systems have further pushed the boundaries of Z-scheme efficiency. A Zn-Pt porphyrin conjugated polymer/BiVO_4_ Z-scheme photocatalytic system, constructed via an *in-situ* Sonogashira coupling reaction, exhibited an AQY of 9.85% at 400 nm for photocatalytic H_2_ evolution.^69^ Furthermore, a rationally designed g-C_3_N_4_/g-C_3_N_4_ Z-scheme heterojunction with tunable boron doping and nitrogen vacancies achieved an impressive AQY of 23.52% at 420 nm and a solar-to-hydrogen (STH) efficiency of 1.16%.^70^ The charge separation efficiency in these artificial photocatalytic systems is fundamentally governed by the driving force of the interfacial IEF. To address the persistent challenges of sluggish interfacial kinetics and low carrier extraction efficiency, modulating the IEF intensity is critical. A quintessential example is the CdTe/V-In_2_S_3_ hybrid photocatalyst. By optimizing the vanadium doping content and CdTe quantum dot (QD) size, researchers successfully upshifted the Fermi level of V-In_2_S_3_ and downshifted that of CdTe, constructing a robust built-in electric field (Figures 4E and 4F). When coupled with a Pt/CoO_x_ cascade co-catalyst system (Figure 4G), this hybrid Z-scheme architecture achieved an extraordinary internal quantum efficiency of 114% and an AQY of 73% at 350 nm, alongside a remarkable STH efficiency of 1.31%.^68^

Despite these remarkable achievements, Z-scheme heterojunctions are frequently constrained by inherent structural limitations. A primary drawback is their reliance on a mediating conductive material—typically noble metals such as Ag or Pt—to facilitate carrier recombination at the interface. The incorporation of noble metals not only escalates material costs but also introduces a detrimental “light-shielding effect”. Because these metallic mediators often possess strong intrinsic light-absorption or scattering capabilities, they competitively parasitize incident photons, resulting in a significant reduction in the actual photon capture efficiency of the adjacent semiconductors.^71^

To definitively resolve these issues, the S-scheme heterojunction has emerged as a superior interfacial architecture. An S-scheme system operates entirely without exogenous electron mediators. It typically comprises a reduction-type semiconductor (characterized by a higher Fermi level and smaller work function) and an oxidation-type semiconductor (with a lower Fermi level and larger work function).^72^^,^^73^ Upon intimate physical contact, spontaneous electron diffusion from the reduction-type to the oxidation-type semiconductor establishes a strong IEF and induces interfacial band bending.^74^ Under illumination, this unique interfacial energy barrier effectively repels the highly reductive electrons and highly oxidative holes, keeping them segregated within their respective original semiconductors. Conversely, it forces the thermodynamically “useless” photogenerated electrons in the oxidation-type semiconductor and holes in the reduction-type semiconductor to rapidly recombine at the interface. Consequently, the S-scheme heterojunction simultaneously maximizes spatial charge separation, preserves the most potent redox-active species, and eliminates parasitic light absorption, establishing itself as the highly effective architecture for advanced photocatalytic design.

Currently, the validation of S-scheme charge transfer has transcended theoretical assumptions, now relying on rigorous *in-situ* and ultrafast mechanistic verification. Advanced characterization techniques—such as *in situ* irradiated X-ray photoelectron spectroscopy (ISI-XPS),^75^^,^^76^ Kelvin probe force microscopy (KPFM),^77^ and femtosecond transient spectroscopy (fs-TAS)^78^—are now the gold standard for dynamically tracking interfacial charge flow. Under *operando* illumination, ISI-XPS can directly observe the binding energy of the reduction-type semiconductor shifting to higher values (indicating electron loss at the interface) while the oxidation-type semiconductor shifts to lower values (indicating electron gain), providing incontrovertible proof of the directed S-scheme charge transfer pathway. Beyond mechanistic verification, the structural design of S-scheme heterojunctions is rapidly advancing toward multi-dimensional and atomic-level precision. At the mesoscopic scale, rational dimensional engineering plays a vital role in maximizing the interfacial contact area. A quintessential strategy involves the construction of 1D/2D heterostructures, such as the coupling of 1D W_18_O_49_ nanowires with 2D porous g-C_3_N_4_ nanosheets.^79^ This architecture affords expansive face-to-face contact interfaces that significantly shorten charge migration distances and accelerate reaction kinetics. Similarly, hierarchical architectures such as the Mn_0.2_Cd_0.8_S@CoAl LDH core-shell heterojunction have been rationally designed to establish a robust and widespread interfacial electric field, effectively directing the S-scheme charge flow for highly efficient H_2_ evolution.^80^

**Figure 5:**
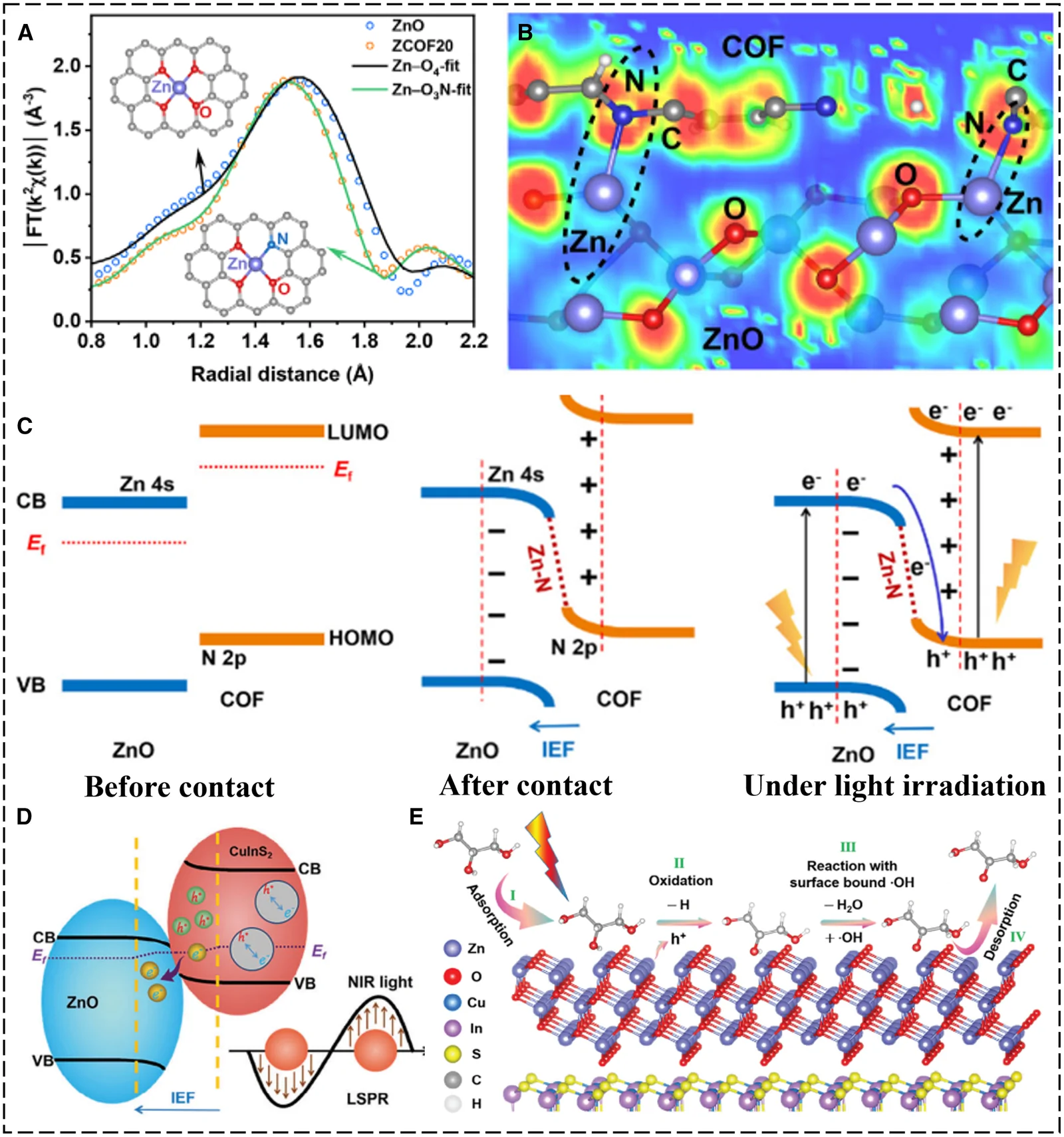
Mechanistic paradigms and atomic-level charge dynamics in S-scheme heterojunctions Atomic-level structural and electronic insights into the S-scheme ZnO/COF interface: (A) structural models of the Zn-O_4_ and Zn-O_3_N coordination environments, (B) 2D electron localization function (ELF) mapping, and (C) schematic of the IEF formation and directed S-scheme charge transfer via interfacial Zn-N bonds under irradiation.^81^ Copyright 2025 Wiley-VCH GmbH. Dual-functional catalytic mechanisms of the ZnO/CuInS_2_ heterojunction: (D) S-scheme charge transfer coupled with the NIR-driven LSPR effect, and (E) the proposed reaction pathway for the selective photooxidation of glycerol to dihydroxyacetone.^82^ Copyright 2024 Wiley-VCH GmbH.

Pushing this boundary to the atomic scale, researchers are now constructing well-defined covalent linkages to bypass the high interfacial resistance of traditional physical blending. A seminal example is the engineering of an S-scheme heterojunction between nanostructured ZnO and an imine-based covalent organic framework (COF) (Figures 5A and 5B). The interfacial Zn─N bonds act as a highly efficient atomic “gateway” for the targeted recombination of carriers, drastically boosting the overall spatial charge separation efficiency (Figure 5C).^81^ Empowered by this uncompromised redox capability, S-scheme heterojunctions are increasingly deployed for challenging, high-value dual-functional reactions that surpass simple degradation or single-half-reaction systems. For instance, Meng et al. ingeniously designed a plasmonic, NIR-responsive ZnO/CuInS_2_ S-scheme photocatalyst (Figure 5D).^82^ The S-scheme charge transfer between the ZnO matrix and CuInS_2_ QDs profoundly alters the interfacial electronic dynamics, transforming the inherently NIR-inactive ZnO into a highly NIR-active composite. This amplified optical response is attributed to the accumulation of free charge carriers at the interface following charge migration, which synergistically enhances the localized surface plasmon resonance (LSPR) effect of the CuInS_2_ QDs. Driven by the robust S-scheme IEF and the accompanying plasmonic photothermal effect, this architecture maximizes atom economy and solar-to-chemical conversion efficiency in complex dual-functional catalytic applications (Figure 5E).

### Cocatalyst-induced additional interface for surface kinetic regulation

While the rational design of S-scheme heterojunctions effectively addresses the spatial separation and thermodynamic preservation of photogenerated carriers, the overall photocatalytic efficiency often remains constrained by a final barrier: sluggish surface reaction kinetics. It is crucial to distinguish the role of noble metals functioning as surface cocatalysts in S-scheme systems from their role as interfacial mediators in conventional Z-scheme architectures.^83^^,^^84^ In mediator-based Z-scheme systems, noble metals are physically sandwiched between two semiconductors, which inevitably induces a severe “light-shielding effect” and parasitically absorbs incident photons. Conversely, in S-scheme heterojunctions, because interfacial charge recombination is thermodynamically driven by the built-in electric field, cocatalysts are exclusively deposited on the outermost surface. This surface configuration not only eliminates light-shielding but also precisely utilizes these sites to fundamentally lower the activation energy of the final redox reactions without negating the advantages of the S-scheme.^71^

To maximize the atom economy of expensive noble metals, recent research has shifted toward the precise atomic-level manipulation of cocatalyst morphologies and deposition sites. A compelling paradigm is demonstrated by the *in situ* photodeposition of Pt clusters onto COFs.^85^ By strategically introducing adjacent hydroxyl groups and imine-N sites within the COF structural units where photogenerated electrons naturally converge, researchers achieved the targeted *in situ* reduction of adsorbed Pt species into highly dispersed metal clusters. This targeted dispersion exposes a massive active surface area and dramatically facilitates electron transfer, culminating in an extraordinary photocatalytic H_2_ evolution rate of 42,432 μmol g^−1^ h^−1^ at a mere 1 wt% Pt loading, highlighting the power of sub-nanometer interfacial structural design.

Given the cost constraints associated with noble metals, the exploration of earth-abundant, highly conductive alternatives has gained immense momentum. 2D materials, particularly transition metal carbides/nitrides (MXene) and transition metal phosphides, have emerged as exceptional noble-metal-free reduction cocatalysts. For instance, mesoporous TiO_2_ nanoparticles anchored onto highly conductive Ti_3_C_2_ MXene nanosheets via electrostatic self-assembly significantly enhanced photo-induced carrier separation. The TiO_2_/Ti_3_C_2_ composite exhibited a 5.6-fold enhancement in H_2_ production alongside near-complete (99.6%) degradation of methyl orange within 40 min.^86^ Similarly, in complex S-scheme architectures, the introduction of non-noble Ni_2_P cocatalysts can profoundly alter carrier dynamics. In a ternary 2D/2D SnNb_2_O_6_/CdS-diethylenetriamine S-scheme system, the decoration of Ni_2_P effectively establishes a directional charge flow (from CdS to SnNb_2_O_6_ and finally to Ni_2_P), pushing the H_2_ evolution performance to an impressive 11,992 μmol g^−1^ h^−1^ without relying on any precious metals.^87^

While reduction cocatalysts efficiently trap electrons for gas evolution, the extraction of sluggish holes via oxidation cocatalysts is equally vital to prevent surface recombination and mitigate photocorrosion. Advanced interfacial engineering now allows for the *in situ* generation of these critical oxidation sites. For example, in an S-scheme heterojunction constructed from graphdiyne (GDY) and CuMn_2_O_4_, photogenerated holes drive the *in situ* exsolution of Mn_2_O_3_ from the CuMn_2_O_4_ matrix. This *in situ* generated Mn_2_O_3_ acts as a robust oxidation cocatalyst, working synergistically with the GDY reduction semiconductor. This engineered spatial migration of electrons and holes to their respective specific active sites suppresses severe recombination, yielding a 13.86-fold enhancement in hydrogen evolution compared to pristine CuMn_2_O_4._^88^

The absolute apex of cocatalyst-interface engineering lies in the synergistic integration of dual cocatalysts (both reduction and oxidation sites) to achieve spatial decoupling. By funneling electrons and holes into entirely isolated reaction microenvironments, researchers can drive highly complex dual-functional reactions simultaneously. Illustrating this pinnacle of design, a dual-cocatalyst system was synthesized by modifying a ZnIn_2_S_4_ matrix with both Ni_2_P and graphene (GR).^89^ In this synergistic platform, GR functions as a highly efficient electron relay station, while Ni_2_P acts as the terminal active site for proton reduction. This spatial decoupling not only maximizes the extraction of photogenerated carriers but successfully couples photocatalytic hydrogen production with the highly selective oxidation of benzyl alcohol (BA). Such strategies chart a transformative new method for combining dual cocatalysts with semiconductor interfaces, simultaneously utilizing photo-induced electrons and holes to harvest clean fuels and high-value chemicals.

### Comparative assessment and synergistic integration of interfacial strategies

For a critical and systematic comparison of the diverse interfacial engineering strategies discussed above, Table 1 summarizes their core mechanisms, driving forces for charge separation, and inherent limitations. This comparison reveals several instructive trends. First, there is a clear evolutionary progression in heterojunction design: Type-II architectures achieve spatial charge separation at the expense of thermodynamic driving force, Z-scheme systems recover this driving force through mediator-assisted recombination but introduce noble-metal dependence and parasitic light shielding, whereas S-scheme heterojunctions resolve both issues by exploiting a mediator-free internal electric field, thereby realizing full preservation of the strongest redox potentials without optical penalty. Second, doping and single-atom interfaces operate primarily at the atomic scale, modulating local electronic structure and polarization, and are therefore most effective when synergistically coupled with heterojunction architectures rather than deployed in isolation. Third, spatially decoupled dual cocatalysts address a distinct bottleneck—surface reaction kinetics—and represent the terminal functional module that converts efficiently separated carriers into desired products with minimal overpotential. Collectively, no single strategy is universally optimal; instead, the highest photocatalytic performance would be achieved by hierarchically integrating multiple interfacial design principles.

**Table 1:** Comparative assessment of representative interfacial engineering strategies in photocatalysis

Strategy	Core mechanism	Driving force for charge separation	Inherent limitation
Bulk/interfacial doping	introduction of impurity levels or polarization centers	local lattice distortion / electronegativity difference	excess doping creates recombination centers; no spatial charge separation
Type-II heterojunction	staggered band alignment → spatial separation of e^−^ and h^+^	band offset	thermodynamic loss of driving force; sluggish kinetics
Z-scheme heterojunction	mediator-assisted recombination of weak carriers, retention of strong ones	Ohmic contact / conductive mediator (e.g., Pt, Ag)	noble-metal cost, light-shielding effect, backward reactions from mediators
S-scheme heterojunction	internal electric field (IEF)-driven recombination of useless e^−^/h^+^ at interface	work function difference → IEF and band bending	requires well-matched work functions and intimate interfacial contact
Single-atom interface	asymmetric coordination creates localized polarization centers for charge trapping and reactant activation	metal-support interaction	low loading limit; long-term stability under redox stress
Spatially decoupled dual cocatalysts	spatial decoupling of reduction and oxidation sites to suppress surface recombination and lower activation barriers	Schottky junction or IEF with the host semiconductor	high synthetic precision required; potential parasitic light absorption

Taken together, these interfacial strategies do not function in isolation but instead constitute a continuous design spectrum. Elemental and single-atom doping tailors the electronic structure at the atomic level, thereby providing highly active single-component building blocks for heterojunction construction. Among the various heterojunction architectures, the S-scheme system currently offers the most favorable thermodynamic-kinetic balance, as it fully resolves the long-standing redox-potential sacrifice of Type-II junctions and the light-shielding effect inherent to conventional Z-scheme mediators. Further pushing this modulation to the atomic limit, single-atom interfaces create precisely defined polarization centers that maximize charge localization. Finally, spatially decoupled dual cocatalysts serve as the terminal executors, directionally funneling the already separated electrons and holes into targeted redox reactions with high efficiency. Looking forward, a hierarchical assembly that synergistically integrates atomic-level doping, an S-scheme heterojunction, and spatially decoupled dual cocatalysts represents the most promising architecture for achieving industrial viability.

Through the synergistic integration of doping engineering, S-scheme heterojunction construction, and spatially decoupled cocatalysts, researchers have successfully established robust physical pathways for efficient charge separation and thermodynamic preservation. However, these exquisite interfacial designs are fundamentally based on static band theories and thermodynamic deductions. A critical scientific gap remains: how can we indisputably prove that photogenerated charges follow these proposed multidimensional pathways during a real catalytic reaction? Traditional “black-box” performance evaluations—such as merely measuring the final hydrogen yield or degradation rate—are no longer sufficient to meet the rigorous standards of contemporary photocatalysis research. To demystify the complex catalytic processes, the research paradigm must decisively shift from static structural design to dynamic *operando* tracking. This necessitates the deployment of cutting-edge *in situ* characterization techniques, ultrafast spectroscopy, and theoretical computational models. Only by visually and quantitatively probing the transient charge transfer kinetics and the evolution of intermediate reactive species at the atomic level can we profoundly decipher the intrinsic mechanism of interfacial photocatalysis, which will be systematically discussed in the following section.

## Intrinsic dynamics of interfacial photocatalysis

### The complete photocatalytic cycle at interfaces

**Figure 6:**
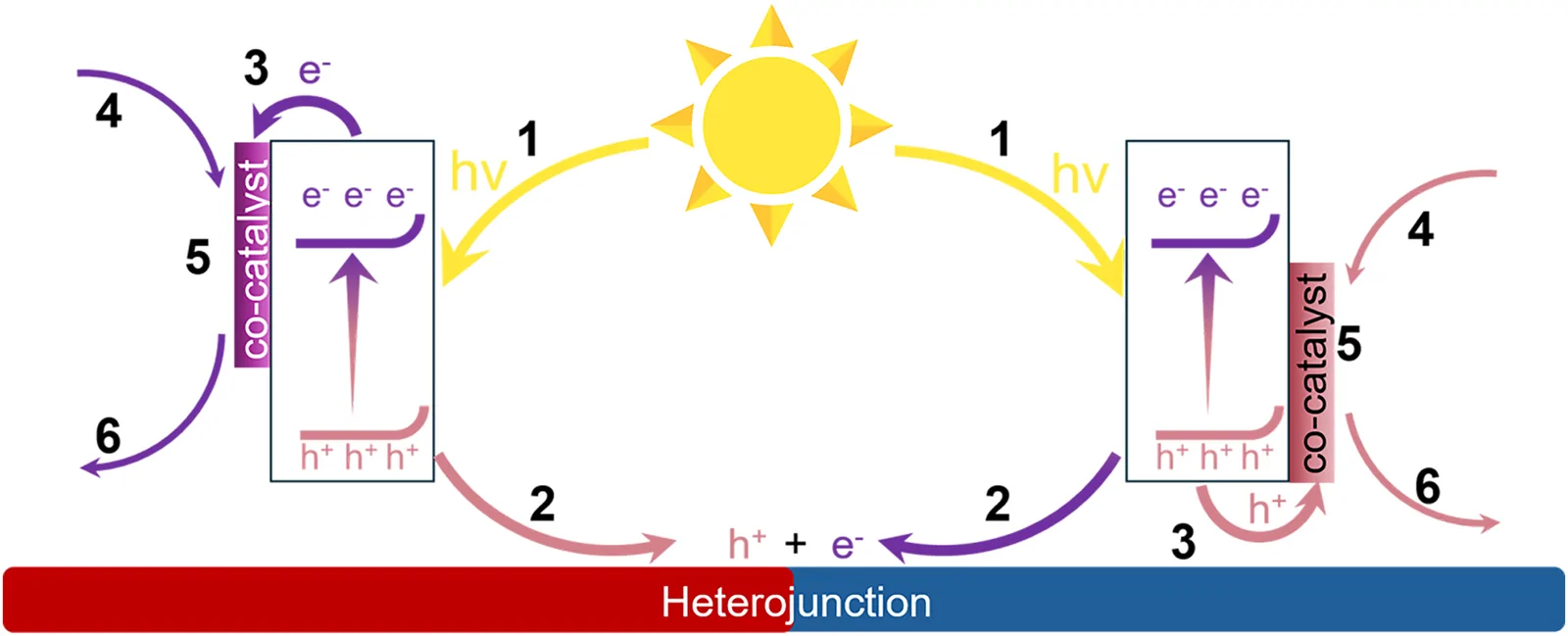
The complete interfacial photocatalytic cycle Schematic illustration of the six fundamental elementary steps spanning from photon harvesting to surface conversion: (1) optical harvesting and exciton generation, (2) bulk carrier migration and interfacial spatial separation, (3) cross-interface migration and localized accumulation, (4) interfacial reactant adsorption and activation, (5) multistep interfacial electron transfer, and (6) product desorption alongside interfacial regeneration.

The overall interfacial photocatalytic process is not an isolated reaction but a complex physicochemical cascade spanning a vast spatiotemporal scale—from femtosecond-level excitation to macroscopic product desorption.^90^ A complete catalytic cycle can be systematically delineated into six fundamental elementary steps (Figure 6).^91^ The cycle initiates with “1. Optical Harvesting and Exciton Generation”. Upon irradiation, photons with energy equal to or exceeding the semiconductor bandgap excite valence band electrons to the conduction band, generating primary electron-hole (e^−^/h^+^) pairs. Interfacial doping or heterojunction construction can significantly broaden the optical absorption edge by introducing impurity levels or narrowing the effective bandgap. Following excitation, the system enters the critical rate-limiting stage: “2. Bulk Carrier Migration and Interfacial Spatial Separation”. Driven by the robust thermodynamic forces of the IEF and interfacial band bending, photogenerated e^−^/h^+^ pairs undergo directed spatial separation, which fundamentally suppresses non-radiative bulk recombination. Rational structural motifs—such as Schottky barriers and S-scheme/Z-scheme heterojunctions—are pivotal for overcoming this kinetic bottleneck.^92^

Subsequently, the separated carriers undergo “3. Cross-Interface Migration and Localized Accumulation”. Electrons are targeted toward reduction cocatalysts (e.g., Pt, CoP), while holes are rapidly extracted by oxidation cocatalysts (e.g., CoO_x_, NiO_x_). This directional funneling establishes highly active, charge-rich localized zones at the respective interfaces.^93^ The cycle then transitions from physical carrier dynamics to surface chemistry during “4. Interfacial Reactant Adsorption and Activation”. Substrate molecules (e.g., H_2_O, CO_2_) chemisorb onto localized defect sites or cocatalyst surfaces. Hole-driven sites facilitate the cleavage of O–H bonds to generate ·OH and O_2_, while electron-driven sites significantly lower the formation energy barriers for critical intermediates (e.g., ∗H, ∗COOH). The specific atomic coordination environment directly dictates the evolutionary pathway and product selectivity. Through “5. Multistep Interfacial Electron Transfer”, the reactants undergo continuous PCET at the active sites to execute the targeted multielectron redox reactions.^94^^,^^95^ The interfacial microenvironment directly governs the macroscopic catalytic kinetics by modulating the activation energy of the rate-determining step (RDS). Ultimately, the cycle concludes with “6. Product Desorption and Interfacial Regeneration”, wherein the target products diffuse into the bulk phase, residual unreacted e^−^/h^+^ pairs undergo surface recombination, and the active sites regenerate to initiate the subsequent cycle.

### Operando tracking of interfacial photocatalysis

Although the six-step mechanism constructs a flawless theoretical framework, relying solely on static band alignments and “black-box” terminal yield measurements is no longer sufficient in contemporary photocatalysis research. The current paradigm has decisively shifted toward the dynamic, *operando* tracking of intrinsic catalytic mechanisms. By integrating state-of-the-art characterization techniques, researchers have established a rigorous “gold standard” for probing both physical charge transfer (steps 1–3) and surface chemical conversions (steps 4–6).

**Figure 7:**
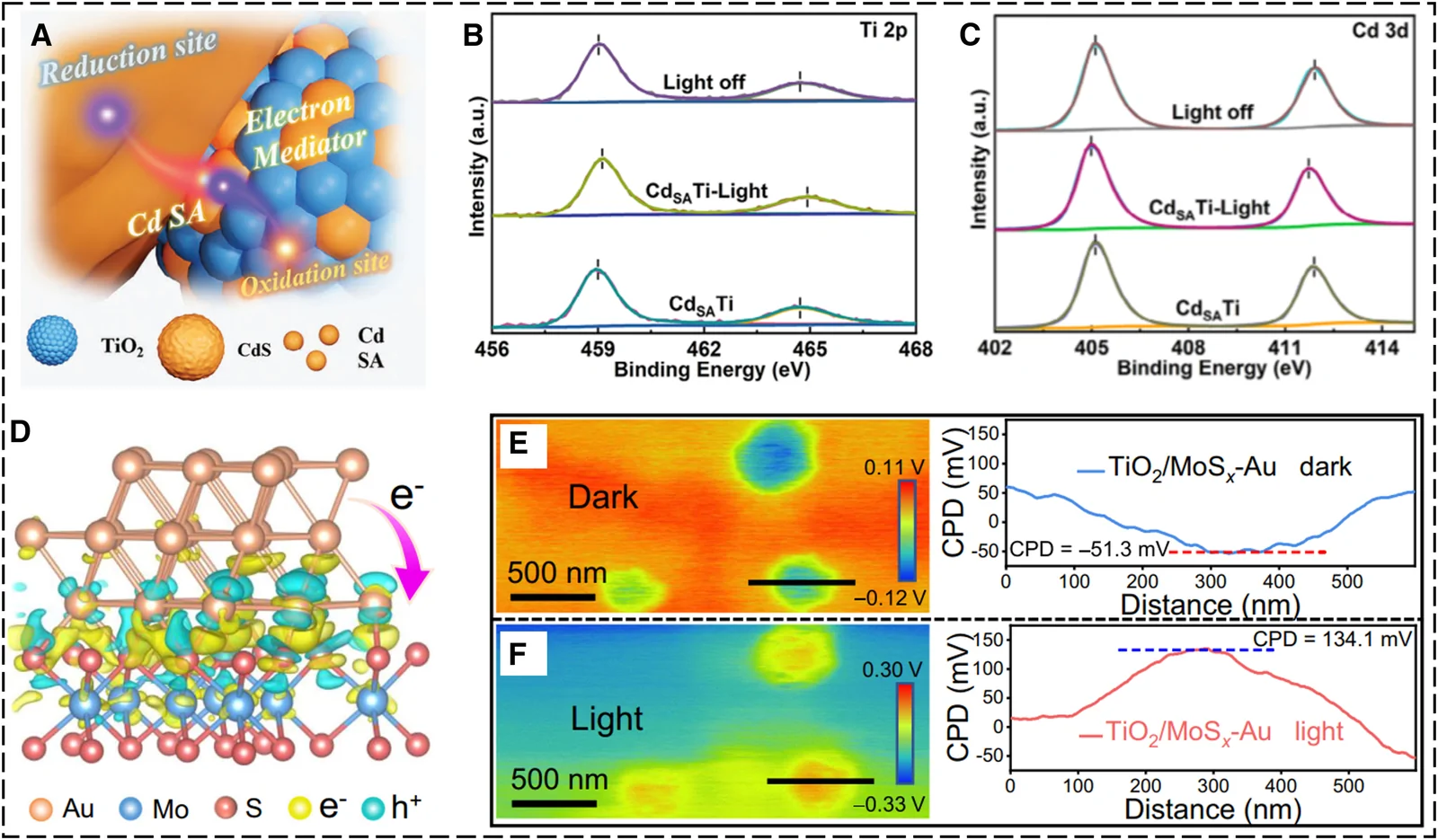
Operando spectroscopic and microscopic verifications of S-scheme charge transfer (A) Schematic of an S-scheme heterojunction. ISI-XPS spectra of the (B) Ti 2p and (c) Cd 3d regions for the Cd_SA_-Ti photocatalyst.^76^ Copyright 2025 Wiley-VCH GmbH. (D) Local charge density difference mapping of MoS_x_-Au (yellow/cyan regions indicate electron accumulation/depletion). (E and F) KPFM images and corresponding surface potential profiles of the TiO_2_/MoS_x_-Au composite (E) in the dark and (F) under 365 nm LED illumination (Scale bars in (E) and (F) represent 500 nm).^96^ Copyright 2024 Springer Nature Ltd.

To indisputably prove IEF-driven directional charge separation—particularly within the highly scrutinized S-scheme mechanism—ISI-XPS has emerged as the widely accepted diagnostic criterion. By monitoring the system under alternating dark and illuminated conditions, ISI-XPS directly captures the transient shifts in binding energies: shifting to higher fields for the reduction-type semiconductor (indicating interfacial electron depletion) and lower fields for the oxidation-type semiconductor (indicating electron accumulation). This real-time fluctuation in extranuclear electron density provides explicit, “fingerprint-level” evidence of cross-interface electron flow. For instance, Ruan et al. proposed a catalyst architecture featuring an S-scheme heterojunction mediated by single Cd atoms, formed through the interfacial coupling of CdS and TiO_2_ nanoparticles.^76^ During ISI-XPS measurements under irradiation, the binding energy of Ti 2p in the illuminated catalyst shifted toward higher energy levels, while that of Cd 3d shifted toward lower energy levels—compared to the catalyst in the dark. This observation indicates a decrease in electron density in the vicinity of Ti 2p and a corresponding increase in electron density surrounding CdS (Figures 7A–7C). These findings suggest the presence of an internal electric field at the interface between TiO_2_ and CdS, which facilitates the transport of photogenerated charge carriers and the construction of the S-scheme heterojunction. Concurrently, KPFM visually maps the contact potential difference (CPD) at the nanoscale, quantitatively verifying the direction and magnitude of interfacial band bending. Zhang et al. reported the synthesis of a TiO_2_/MoS_x_-Au interfacial composite photocatalyst, which exhibited a significant increase in its CPD value under illumination (an increase of approximately 185.4 mV—specifically, rising from −51.3 mV to 134.1 mV).^96^ Concurrently, owing to the enhanced accumulation of holes on the TiO_2_ surface, the color of the material shifted from blue to red (Figures 7D–7F). This phenomenon strongly indicates that photogenerated electrons were efficiently transferred from the TiO_2_ to the MoS_x_-Au co-catalyst.

**Figure 8:**
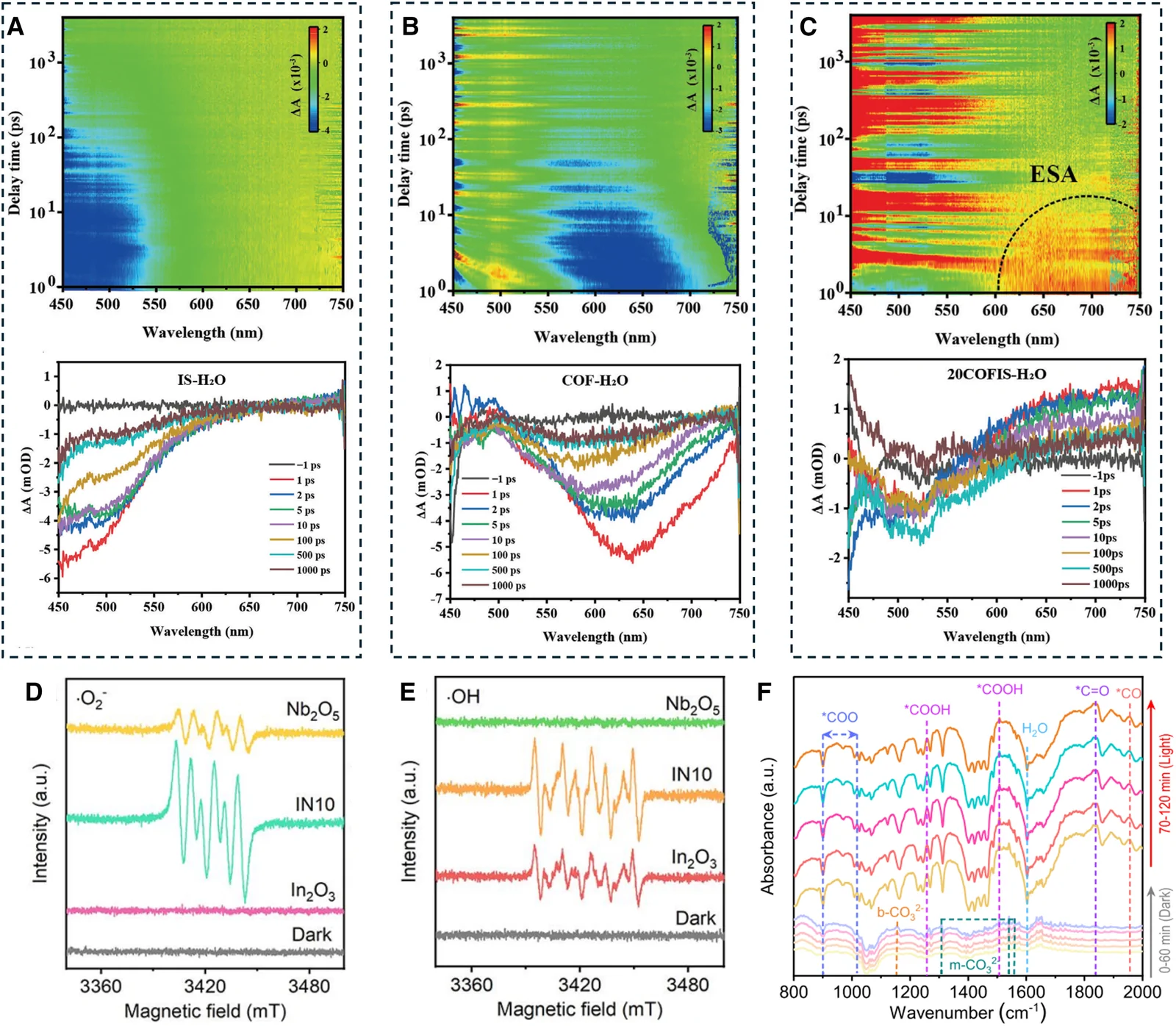
Operando characterizations of transient charge dynamics and interfacial surface reactions 2D pseudocolor fs-TA plots and corresponding spectra of (A) pristine In_2_S_3_ (IS), (B) pristine COF, and (C) the COF/In_2_S_3_ composite (20COFIS), acquired under 400 nm laser pulse excitation in aqueous media.^78^ Copyright 2024 Wiley-VCH GmbH. EPR spectra of (D) DMPO-·O_2_^–^ and (E) DMPO-·OH spin adducts generated over In_2_O_3_, Nb_2_O_5_, and the In_2_O_3_/Nb_2_O_5_ (IN10) heterojunction. (F) *in-situ* FTIR spectra tracking the dynamic photocatalytic CO_2_ reduction process over the IN10 interface.^97^ Copyright 2024 Springer Nature Ltd.

To vividly illustrate the power of ultrafast spectroscopy in unraveling interfacial charge dynamics and electronic coupling, a compelling case study is the organic/inorganic COF/In_2_S_3_ S-scheme heterojunction recently developed by Qiu, Yu, and co-workers. They employed fs-TAS to comprehensively track the sub-nanosecond carrier evolution within the composite (Figures 8A–8C).^78^ For the pristine materials, the fs-TAS of the isolated COF and IS exhibit their characteristic ground-state bleaching (GSB) and excited-state absorption (ESA) signals, which are governed by intrinsic bulk recombination kinetics. Strikingly, upon the *in situ* hybridization of IS nanosheets onto the COF substrate, the 2D pseudocolor TA plots and spectral features of the composite undergo a profound dynamic evolution. This interfacial electronic hybridization not only significantly bridges the energy gap to broaden visible-light absorption but also establishes a high-speed kinetic channel. This direct, ultrafast visualization provides incontrovertible proof of the S-scheme charge transfer mechanism, elucidating how precise interfacial atomic coordination empowers the composite with exceptional redox capacity for highly efficient, pure-water H_2_O_2_ photosynthesis.

Once the carriers enrich on the surface, elucidating the substrate activation pathway becomes the core of mechanistic inquiry. Electron paramagnetic resonance (EPR) spectroscopy, coupled with spin-trapping agents, serves as the premier technique for dynamically monitoring the transient ROS (e.g., ·OH, ·O_2_^-^) generated during Step 4. Furthermore, to thoroughly decipher the complex PCET pathways in Step 5, *in situ* Fourier transform infrared (FTIR) spectroscopy demonstrates irreplaceable value. Under true *operando* conditions, *in situ* FTIR continuously tracks the dynamic processes of reactant adsorption, bond cleavage, and new bond formation. By capturing the vibrational signatures of key surface intermediates (such as the ∗COOH or ∗CHO radicals in CO_2_ reduction), researchers can chart the elementary reaction coordinates with unprecedented precision. Deng et al. employed spin-trapping EPR to trace the spatial accumulation of photogenerated carriers in an In_2_O_3_/Nb_2_O_5_ S-scheme heterojunction. The formation of specific spin adducts utilizing 5,5-dimethyl-1-pyrroline N-oxide (DMPO) inherently demands stringent thermodynamic driving forces: generating the DMPO–·O_2_^−^ adduct requires a reduction potential of –0.74 V (vs. SHE), while the DMPO–·OH adduct requires an oxidation potential of +2.28 V. While the pristine In_2_O_3_ and Nb_2_O_5_ counterparts exhibited sluggish radical generation, the In_2_O_3_/Nb_2_O_5_ composite demonstrated profoundly amplified EPR signals for both ·O_2_^−^ and ·OH radicals under identical illumination conditions (Figures 8D and 8E).^97^ FTIR spectroscopy allowed for the direct observation of the intricate CO_2_ reduction pathway (Figure 8F). Initially, under dark conditions, the introduction of CO_2_ into the system triggered the immediate emergence of vibrational bands assigned to bidentate (b-CO_3_^2−^) and monodentate (m-CO_3_^2−^) carbonates. This serves as explicit evidence for the spontaneous chemisorption and structural activation of the linear CO_2_ molecules at the interfacial active sites (corresponding to Step 4 of the catalytic cycle). Strikingly, upon light irradiation, a dynamic cascade of new transient vibrational signatures rapidly evolved. The *in situ* detection of key intermediates—specifically the carboxyl radical ∗COOH (1258 and 1507 cm^−1^), ∗COO (900 and 1017 cm^−1^), carbonyl ∗C=O (1839 cm^−1^), and adsorbed ∗CO (1956 cm^−1^)—provided a real-time, visual readout of the PCET process.

Ultimately, to seamlessly close the mechanistic loop, these empirical observations captured via *operando* spectroscopy must be fundamentally corroborated by density functional theory (DFT) calculations. By constructing precise interfacial atomic models, DFT extracts the charge density difference to theoretically validate the macroscopic spatial separation observed via XPS. More critically, by calculating the Gibbs-free energy (Δ G) for various reaction pathways in Steps 4 and 5, theoretical simulations decode exactly how meticulously engineered atomic sites or cocatalysts lower thermodynamic activation barriers. This integration bridges macroscopic catalytic performance with quantum-level interfacial dynamics, forming an impenetrable logical loop.

## Recent advances in the applications of interfacial photocatalysis

### Photocatalytic hydrogen evolution

The conversion of abundant solar energy into clean, storable hydrogen fuel via photocatalytic water splitting represents a cornerstone of sustainable energy research.^8^^,^^98^ Focusing specifically on the reductive half-reaction, the generation of molecular hydrogen relies on the efficient extraction and utilization of photogenerated electrons to drive the complex PCET process (2H^+^ + 2e^−^→H_2_).^25^^,^^99^ Thermodynamically, this requires that the CBM of the photocatalyst must be positioned at a more negative potential than the standard proton reduction potential (0 V vs. NHE).^100^^,^^101^ However, achieving high-efficiency hydrogen evolution in single-component semiconductors is severely hampered by sluggish surface reaction kinetics, substantial activation overpotentials, and the ultrafast recombination of electron-hole pairs.^9^^,^^102^^,^^103^^,^^104^^,^^105^^,^^106^ Advanced interfacial engineering, particularly the rational construction of S-scheme heterojunctions and the spatial integration of tailored cocatalysts, provides a definitive solution to these bottlenecks. Unlike conventional Type-II architectures that inevitably sacrifice redox capability, advanced interfacial designs thermodynamically preserve the highest-energy electrons exclusively within the CBM of the reduction-type semiconductor. Driven by the robust IEF, these highly reactive electrons are directionally channeled toward specific surface-active sites or spatially decoupled cocatalysts. These localized interfacial domains function as high-density “electron sinks”, effectively lowering the thermodynamic energy barrier for intermediate proton adsorption (∗H) and facilitating the rapid desorption of H_2_ molecules. Recent cutting-edge studies perfectly exemplify how modulating these specific interfacial parameters translates to macroscale performance breakthroughs across diverse systems.

**Figure 9:**
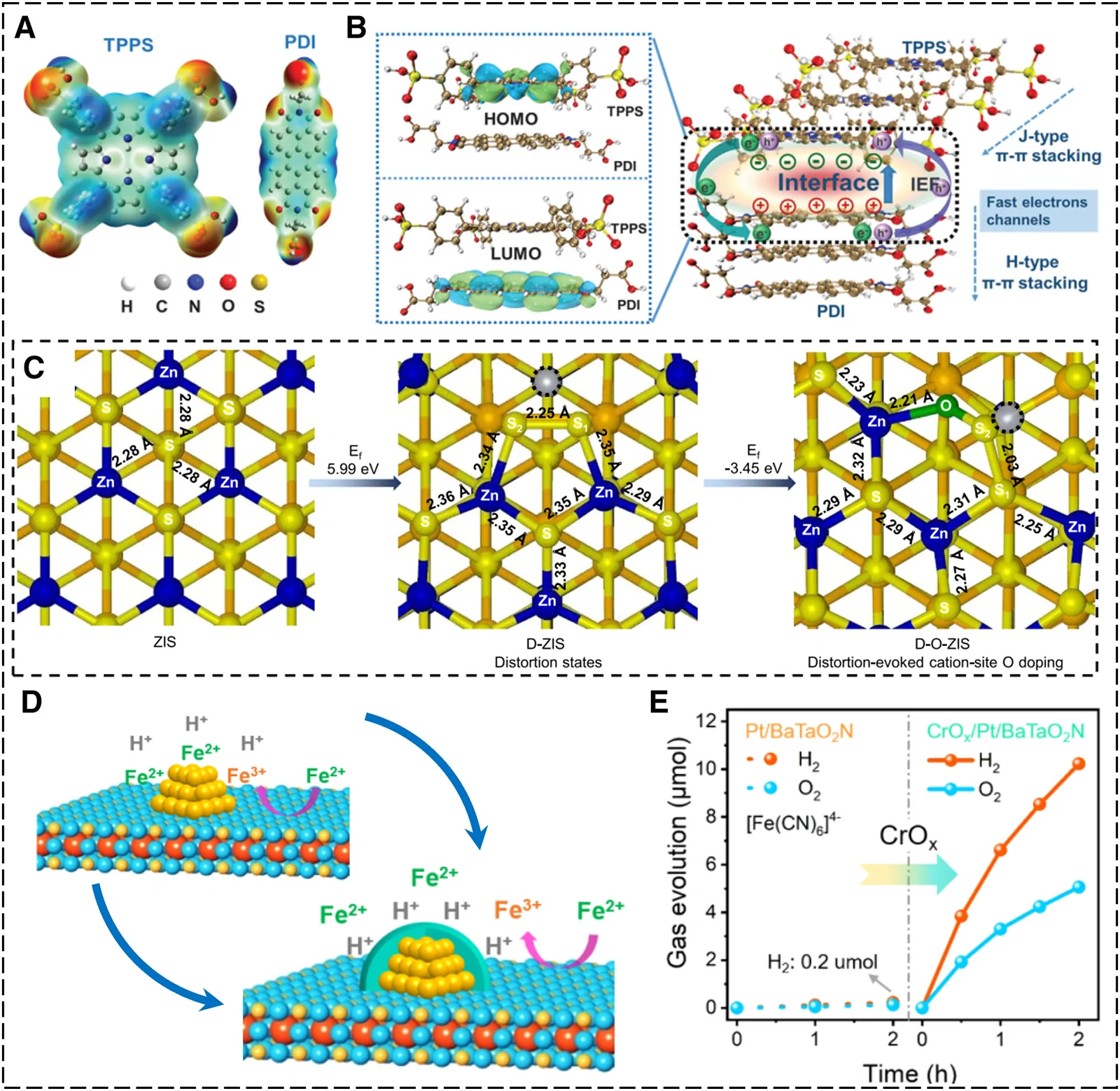
Interfacial molecular engineering and surface shielding strategies for enhanced photocatalytic hydrogen evolution (A) Chemical structures and electrostatic potential distributions of TPPS and PDI molecules. (B) Frontier molecular orbital alignments at the interface (left) and schematic model illustrating the interfacial interactions within the co-assembled supramolecular TPPS/PDI heterojunction (right).^31^ Copyright 2022 Wiley-VCH GmbH. (C) Schematic depicting the local structural transformation, lattice distortion, and cation-site O-doping in the D-*O*-ZIS matrix, with corresponding bond lengths highlighted.^107^ Copyright 2024 Springer Nature Ltd. (D) Schematic illustration of the altered adsorption behavior and suppressed backward reactions of redox shuttle ions on the cocatalyst surface before and after CrO_x_ encapsulation. (E) Photocatalytic Z-scheme water-splitting performance of various HER systems under visible-light irradiation (λ ≥ 420 nm).^108^ Copyright 2025 American Chemical Society.

To fundamentally overcome the kinetic sluggishness of the HER, maximizing the thermodynamic driving force for charge separation is paramount. Constructing organic electron D-A interfaces has proven highly effective in this regard. For instance, a meticulously designed D-A interface between tetra(4-sulfonatophenyl) porphyrin (TPPS) and perylene diimide (PDI) generated a giant internal electric field—approximately three to four times stronger than those of the pristine components (Figures 9A and 9B).^31^ This amplified built-in electric field profoundly facilitated spatial charge separation and significantly prolonged the excited-state lifetime of the carriers. Consequently, the TPPS/PDI composite delivered an exceptional H_2_ evolution rate of 30.36 mmol h^−1^ g^−1^, nearly an order of magnitude higher than its single-component counterparts, highlighting the decisive role of interfacial polarization. To further amplify this strategy, researchers have leveraged molecular dipole engineering to construct D-A supramolecular architectures capable of full-spectrum light harvesting. A pioneering paradigm is the TPPS/C_60_ supramolecular photocatalyst, which exhibits an exceptionally broad optical response (300–850 nm) with a theoretical spectral utilization efficiency of up to 70%.^109^ Driven by the enhanced molecular dipoles within the interleaved architecture, a giant IEF is established, effectively capturing a remarkably long-lived charge-separated state. This drastically retards radiative recombination and elevates the charge-separation efficiency by a factor of 2.35, culminating in an extraordinary H_2_ evolution rate of 34.57 mmol h^−1^ g^−1^. Beyond initial charge separation, maintaining long-term catalytic stability remains a critical challenge, particularly for narrow-bandgap metal sulfides (e.g., CdS) that are notoriously plagued by severe photo-corrosion induced by accumulated holes. To bypass this, the spatial decoupling of reductive and oxidative sites via dual-cocatalyst integration is a highly effective strategy. An elegant example is the ternary composite constructed via the *in situ* growth of MoS_2_ and Ti_3_C_2_ MXene on a CdS matrix.^110^ Both experimental characterizations and DFT calculations unequivocally demonstrated a directional charge routing mechanism: photogenerated electrons efficiently migrated to MoS_2_ to drive rapid proton reduction, while photogenerated holes were timely extracted by the highly conductive Ti_3_C_2_ MXene. This tailored spatial segregation completely shielded the CdS lattice from hole-induced self-oxidation. As a result, the optimized composite not only achieved a high H_2_ production rate of 14.88 mmol h^−1^ g^−1^ but also maintained structural integrity over 78 hours of continuous operation, paving the way for the industrial deployment of robust sulfide-based systems.

Pushing interfacial regulation to the atomic scale allows researchers to resolve the inherent instability and functional limitations of traditional photocatalysts. A groundbreaking paradigm is the distortion-evoked cation-site oxygen doping in a ZnIn_2_S_4_ (D-*O*-ZIS) matrix.^107^ The precise substitution of oxygen triggers significant electronegativity differences between adjacent atomic sites, creating a unique asymmetric local structure: electron-rich S_1_ sites and electron-deficient S_2_ sites (Figure 9C). This intense atomic-level charge redistribution enables the S_1_ sites to exquisitely favor the adsorption and desorption of hydrogen intermediates, while the S_2_ sites activate stable oxygen-evolution reactions, completely circumventing the common issue of sulfur lattice instability. Benefiting from this atomic precision, the D-*O*-ZIS system not only sustained robust HER but also successfully unlocked unassisted overall water splitting with a remarkable STH conversion efficiency of 0.57% and a 91% retention rate over 120 hours. While the strategies excel in pure or sacrificial systems, particulate water splitting in Z-scheme architectures utilizing redox mediators (shuttle ions) encounters a unique, long-standing bottleneck.

High-efficiency catalysts often suffer a massive performance drop in mediator solutions due to the strong competitive adsorption of shuttle ions on the metal cocatalyst surface, which poisons the initial proton reduction sites and triggers a severe backward reaction of the oxidized mediators. To systematically address this, researchers developed a highly selective surface modification strategy, depositing amorphous chromium oxide (CrOx) species onto typical metal cocatalysts (e.g., Pt, Ru) supported on BaTaO_2_N and SrTiO_3_:Rh (Figure 9D). The CrO_x_ overlayer acts as an intelligent semi-permeable shield: it substantially weakens the interaction between the metal active sites and the bulky shuttle ions while simultaneously promoting the adsorption of tiny protons for HER. By effectively suppressing the backward reaction, this surface modification enhanced the photocatalytic HER activity by one to two orders of magnitude in diverse mediator solutions, providing a universally feasible strategy for constructing highly efficient Z-scheme water-splitting systems (Figure 9E).^108^

In summary, the strategic modulation of interfacial microenvironments—spanning from supramolecular dipole engineering and atomic-level polarization to intelligent physical shielding—has fundamentally dismantled the long-standing thermodynamic and kinetic barriers of photocatalytic hydrogen evolution. By establishing highly directional and spatially decoupled charge-routing pathways, these rationally designed interfaces guarantee the maximal utilization of high-energy electrons for targeted proton reduction while strictly protecting the catalytic matrix from self-oxidation. Correspondingly, their yield for producing hydrogen was significantly increased, as summarized in Table 2. This exquisite atomic- and molecular-level control over interfacial charge dynamics will not only propel the commercial viability of robust overall water-splitting systems but also serve as a universal architectural blueprint for conquering even more demanding multi-electron catalytic cascades, such as the selective reduction of CO_2_ and the dual-functional upcycling of recalcitrant wastes.

**Table 2:** Comparison of photocatalytic hydrogen evolution performance across various state-of-the-art interfacial photocatalysts

Catalyst	Efficiency μmol g^−1^ h^−1^	External cocatalyst	Sacrificial agents	Light source	Reference
g-C_3_N_4_/ZnIn_2_S_4_	∼20738	Pt	triethanolamine	visible light	Chen et al.^37^
TPPS/PDI	∼30360	Pt	ascorbic acid	visible light	Yang et al.^31^
MO@ZIS	∼33290	Pt	triethanolamine	magnetic field + photothermal	Hao et al.^33^
Ni/MCS-s	∼164100	No	Na_2_SO_3_ and Na_2_S	visible light	Cheng et al.^32^
Ce/N-SrTiO_3_	∼4280	Pt	methanol	visible light	Wang et al.^46^
Bi-OZIS	∼170.22	No	–	visible light	Tang et al.^53^
CdS@MoS_2_/Ti_3_C_2_	∼14880	No	lactic acid	visible light	Wu et al.^110^
Pt1/def-TiO_2_	∼52720	No	methanol	visible light	Chen et al.^111^
Sc-TiO_2_	∼758	Pt	–	visible light	Qin et al.^112^
Ni_2_P-SNO/CdS-D	∼11,992	No	Na_2_SO_3_ and Na_2_S	visible light	Hu et al.^87^
Co_3_O_4_@ZnIn_2_S_4_	∼18900	No	triethanolamine	AM 1.5G sunlight	Shi et al.^113^

### Selective photocatalytic CO_2_ reduction

Mimicking natural photosynthesis to catalytically convert the greenhouse gas CO_2_ into value-added carbonaceous fuels (e.g., CO, CH_4_, and C_2_H_4_) represents the ultimate paradigm toward achieving a closed-loop carbon-neutral cycle.^114^^,^^115^ However, the linear CO_2_ molecule exhibits exceptional thermodynamic stability (with a C=O bond dissociation energy of ∼805 kJ/mol), and its multi-electron PCET pathways are notoriously complex.^116^^,^^117^ More severely, in aqueous media, the CO_2_ reduction reaction (CO_2_RR) must vigorously compete with the kinetically more favorable HER. Consequently, achieving highly efficient directional activation of CO_2_ molecules and precise product selectivity via delicate interfacial engineering constitutes the core challenge in this field. As systematically established by the mechanistic and *operando* characterization frameworks discussed in Section intrinsic dynamics of interfacial photocatalysis, breaking these intrinsic bottlenecks strictly relies on the atomic-level modulation of the interfacial microenvironment.

**Figure 10:**
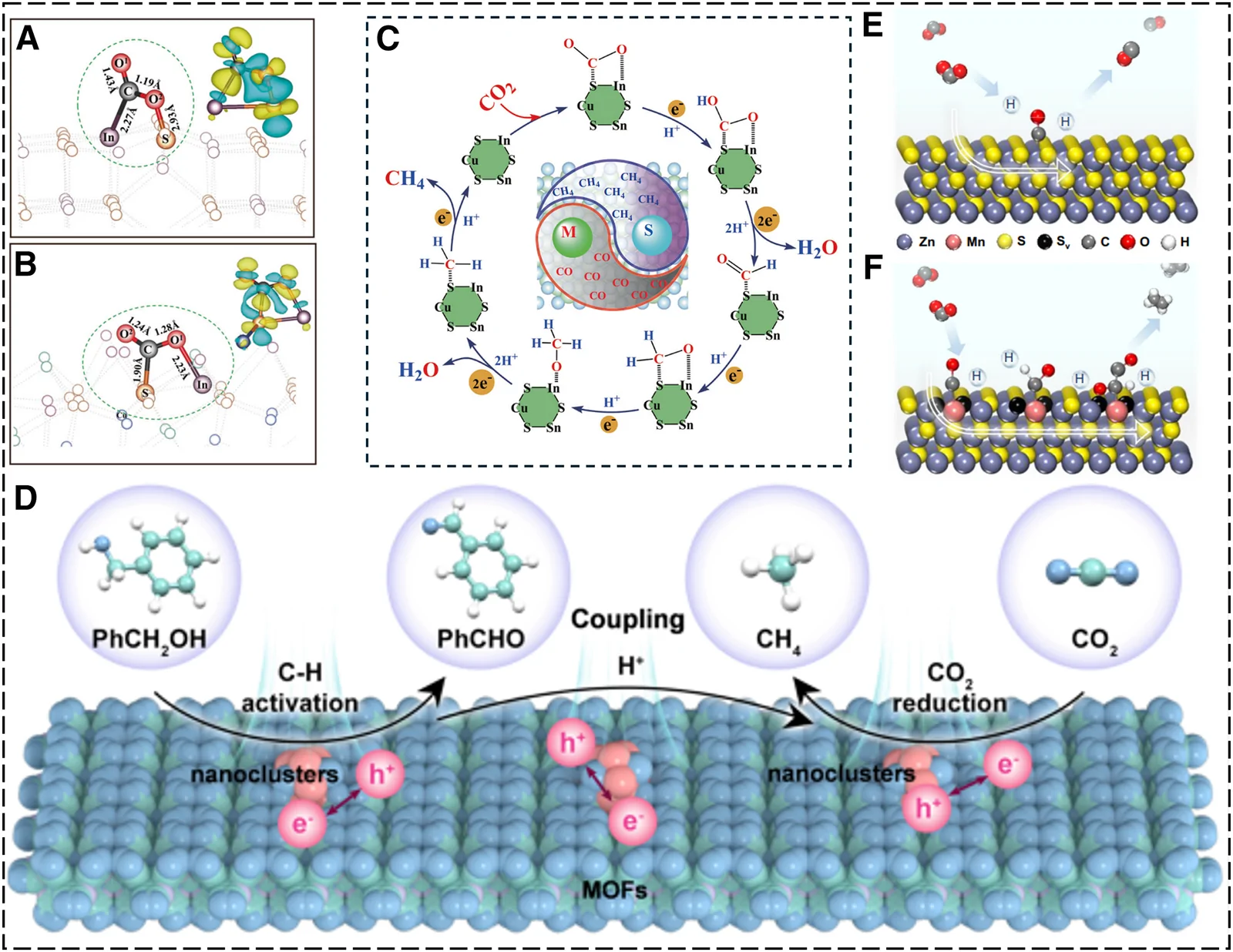
Tailoring interfacial microenvironments for selective photocatalytic CO_2_ reduction and coupled organic synthesis Optimized CO_2_ adsorption configurations and corresponding differential charge density maps (yellow and blue regions denote electron accumulation and depletion, respectively) on (A) pristine In_2_S_3_ and (B) CuInSnS_4_. (C) Proposed catalytic pathway for CO_2_ reduction over the CuInSnS_4_ interface.^119^ Copyright 2023 Springer Nature Ltd. (D) Schematic of the asymmetric dual-metal interface designed for coupling CO_2_-to-CH_4_ conversion with concurrent C–H bond activation.^120^ Copyright 2026 American Chemical Society. Calculated Gibbs free energy diagrams and reaction pathways for CO_2_ methanation over (E) pristine ZnS and (F) the Mn1-ZnSv single-atom interface.^121^ Copyright 2026 Springer Nature Ltd.

Recent pioneering studies demonstrate that such tailored interfaces play a decisive role in balancing the thermodynamic and kinetic requirements of CO_2_RR. The critical first step in breaking the structural symmetry of linear CO_2_ molecules relies on robust electron injection at the interface. To overcome substantial charge transfer resistance and sluggish kinetics, Liu et al. ingeniously constructed a 2D-2D Ohmic-contact heterojunction between oxygen-vacancy-modified bismuth molybdate nanosheets (BMOVs) and 2D bismuthene (Bi/BMOVs). Experimental characterizations and DFT simulations confirmed that the tunable IEF originating from this Ohmic contact profoundly optimizes interfacial charge dynamics. Consequently, the optimized composite achieved nearly 10-fold enhancement in CO_2_-to-CO and CH_4_ conversion rates compared to pristine BMO.^77^ Concurrently, to sustain continuous and efficient electron injection, Sayed et al. proposed a novel concept of an “ionized cocatalyst”. They engineered core-triple shell Mn,C-codoped ZnO hollow spheres, wherein the embedded Mn ions with switchable valence states significantly promote CO_2_ chemisorption and light harvesting. Acting as dynamic electron relay stations, these variable-valence Mn ions continuously capture photogenerated electrons from the conduction band of ZnO and shuttle them to the adsorbed CO_2_ molecules, thereby achieving sustained and highly selective CO_2_ photoreduction.^118^ The deep reduction of CO_2_ to CH_4_ involves a formidable eight-electron transfer barrier, which is highly sensitive to the adsorption configuration and binding strength of reaction intermediates at active centers. A recent study by Chai et al. subverted the conventional paradigm that attributes catalytic activity solely to metal atoms or structural defects. They discovered that within CuInSnS_4_ octahedral nanocrystals with exposed (111) facets, the electron-deficient non-metal sulfur atoms act as the genuine active centers dictating CH_4_ selectivity (Figures 10A–10C). The preferential adsorption of the carbon atom of CO_2_ onto these electron-poor S sites tremendously stabilizes the critical intermediates, successfully switching the product distribution from CO (typically observed on individual metal sulfides such as In_2_S_3_, SnS_2_, and Cu_2_S) to CH_4._^119^

To address the pervasive issue of hole wastage in conventional half-reactions, Song et al. anchored semiconductor nanoclusters within vacancy-rich metal-organic frameworks to construct unique asymmetric dual-metal catalytic interfaces. This tailored asymmetric configuration seamlessly couples the solar-driven reduction of CO_2_ to CH_4_ with the selective oxidative activation of C–H bonds in biomass-derived alcohols (e.g., benzyl alcohol). This tandem reaction strategy not only maximizes the comprehensive utilization of photogenerated electron-hole pairs but also simultaneously yields high-value chemicals, offering a compelling blueprint for dual-functional photocatalysis (Figure 10D).^120^ Among all CO_2_RR products, the generation of multi-carbon (C_2_^+^) compounds such as C_2_H_4_ represents the ultimate “holy grail”, as it strictly requires efficient C-C coupling between two adjacent surface intermediates (e.g., ∗CO and ∗CHO). Tang et al. achieved near-unity photocatalytic conversion of CO_2_ to ethylene by embedding low-coordination manganese single atoms into the sulfur vacancies of a zinc sulfide matrix (Mn_1_-ZnS_v_). *In situ* spectroscopic analyses and DFT calculations revealed that the coordinatively unsaturated, asymmetric Mn-S_3_ configuration induced by sulfur vacancies precisely modulates the local charge distribution. This asymmetric microenvironment not only strengthens ∗CO adsorption but also dramatically lowers the activation energy for the coupling of ∗CO and ∗CHO into the key ∗COCHO intermediate, thereby clearing a high-speed kinetic pathway for C-C coupling (Figures 10E and 10F).^121^ In parallel, dynamic interfacial reconstruction under real reaction conditions offers unique opportunities to reshape reaction pathways. Ren et al. designed a Ru_0.6_Cu_1_/CeO_2_ catalyst featuring adaptive O_2_ tolerance. Driven by the photothermal effect, this system underwent O_2_-mediated dynamic interfacial reconstruction, self-assembling a Cu^0^-Cu^+^ interface with a distinct charge gradient within the Ru/Cu alloy clusters. The synergistic interplay between this unique interfacial charge cascade transfer and specific geometric sites optimized the adsorption behavior of ∗CHOCO intermediates and reconfigured the C-C coupling pathway, successfully redirecting the product selectivity from C_1_ compounds to C_2_H_4_.^122^

In summary, through the precise modulation of interfacial charge dynamics, artificial photocatalytic systems have achieved remarkable milestones in driving efficient hydrogen evolution and highly selective CO_2_ conversion. Table 3 compares the selective photocatalytic CO_2_ reduction performance of different types of interfacial photocatalysts. The thermodynamic preservation of strong redox potentials inherent to S-scheme heterojunctions, coupled with the rational integration of low-coordination single atoms, non-metal active sites, and spatially decoupled dual-cocatalysts, has successfully broken the multi-electron transfer barriers of these challenging reactions. This progression from fundamental charge routing to targeted molecular activation firmly establishes a robust transition from physical carrier separation to precise surface chemistry. Nevertheless, to bridge the immense gap between bench-scale laboratory breakthroughs and commercial industrial deployment, future research must confront and resolve several critical scientific and engineering challenges. (1) The vast majority of current high-performance metrics are strictly limited to micro-scale particulate suspension batch reactors. Future efforts must shift toward large-scale continuous material fabrication techniques (e.g., 3D printing and roll-to-roll coating) to develop innovative flow-through photocatalytic reactor systems characterized by minimized mass-transfer resistance and maximized photon penetration depth.^135^ (2) Industrial flue gases containing high concentrations of O_2_ and SO_2_, alongside complex aqueous matrices featuring high salinity and broad pH fluctuations, easily poison active surface sites or induce acute photocorrosion. Future interfacial designs must incorporate adaptive dynamic reconstruction mechanisms or intelligent protective overlayers (analogous to the CrO_x_ shielding mechanism) to sustain industrial-grade operational stability exceeding thousands of hours.^136^ (3) Given the astronomically vast parameter space of asymmetric coordination environments and multi-component interfaces, traditional trial-and-error experimental paradigms are highly inefficient. Future research should deeply integrate machine learning algorithms with high-throughput computational screenings to map quantitative composition-structure-activity relationship (QSAR) networks, enabling the precise prediction and atomic-level tailoring of specific PCET rate-determining step barriers.^137^^,^^138^

**Table 3:** Comparison of selective photocatalytic CO_2_ reduction performance over various multiscale interfacial photocatalysts

Catalyst	Main products	Light source	Efficiency μmol g^−1^ h^−1^	Reference
BiOCl/In_2_O_3_	CO	300 W Xenon lamp	∼6.9	Liu et al.^123^
co-doped In_2_O_3_	CO	300 W xenon lamp	∼152	Yu et al.^124^
In-plane In_2_O_3_/ZnIn_2_S_4_	CO	300 W Xe lamp with a 400 nm longpass cutoff filter	∼5624	Zhao et al.^125^
CuCoAl-LDHs	CO	300WXe lamp with BUV bulb (380 ≤ λ ≤ 800 nm)	∼73.21	Fan et al.^126^
BiOIO_3_/Zn-MOF-15	CO	300 W xenon lamp	∼58.82	Yuhong et al.^127^
CoTiO_3_/g-C_3_N_4_	CO	300 W Xe lamp	∼43.5	Yang et al.^128^
Ga-CoS_2_	C_2_H_4_	300 W Xe lamp (λ ≥ 420 nm)	∼12.45	An et al.^129^
MoS_2_@COF-15	C_2_H_6_	300 W xenon lamp with a 420 nm-cut filter	∼56.2	Yang et al.^130^
MoS_2_@TPPy-COF	HCOOH	400 W medium-pressure mercury lamp	∼322	Kumar et al.^131^
PDA-TTA	HCOOH	300 W Xe lamp (420 nm ≤ λ ≤ 800 nm)	∼65.7	Jia et al.^132^
CuSe@BiOI	HCOOH	AM 1.5G illumination (I0 = 150 mW cm^−2^, 380 ≤ λ ≤ 780 nm)	∼26.7 μmol cm^− 2^ h^−1^	Gong et al.^133^
d-UiO-66/MoS_2_	CH_3_COOH	300 W Xe-arc lamp with UV cutoff filter (λ > 400 nm)	∼39.0	Yu et al.^134^

### Photocatalytic methane activation and selective conversion

Beyond CO_2_ reduction, the direct photocatalytic conversion of methane (CH_4_) into value-added chemicals under mild conditions has emerged as a rapidly developing frontier that holds immense promise for sustainable chemical synthesis.^139^ Methane, the principal component of natural gas, possesses an exceptionally stable tetrahedral structure with a C–H bond dissociation energy of ∼439 kJ mol^−1^, making its selective activation a formidable thermodynamic and kinetic challenge.^140^^,^^141^ Furthermore, the targeted products (e.g., methanol, ethane, ethylene) are inherently more reactive than CH_4_ itself, rendering them susceptible to undesired over-oxidation to CO_2_ once formed. Consequently, achieving efficient CH_4_ conversion with high product selectivity demands precise control over both the initial C–H bond cleavage and the subsequent reaction pathway—a task for which interfacial photocatalysis is uniquely suited.^142^^,^^143^

Heterojunction engineering plays a pivotal role in meeting the stringent requirements of CH_4_ activation. The one-electron oxidation of CH_4_ to the methyl radical (·CH_3_) requires a redox potential of approximately +2.06 V (vs. NHE), which exceeds the valence band maximum (VBM) of many conventional semiconductors.^144^^,^^145^ S-scheme and Z-scheme heterojunctions are particularly well-suited to address this limitation by spatially decoupling the oxidation and reduction sites while preserving the strongest thermodynamic driving force at each site. A compelling demonstration of the S-scheme strategy is the defect-engineered Def-CuCN/NU heterojunction, which integrates Cu SA anchored on polymeric carbon nitride (CuCN) into defective NH_2_-UiO-66 (Def-NU). In this architecture, the S-scheme charge transfer pathway between CuCN and Def-NU accelerates the separation and transport of photogenerated carriers, while the defective MOF, with its porous structure and abundant active sites, functions as a microreactor that enhances CH_4_ adsorption, facilitates CH_3_OH desorption, and minimizes the contact of methanol with ·OH radicals, thereby effectively suppressing overoxidation. The Cu SA sites further promote the selective activation of O_2_ to ·OOH species rather than the highly oxidative ·OH radicals. Consequently, under full-spectrum light irradiation, the Def-CuCN/NU catalyst achieved an outstanding production rate of 1718 μmol g^−1^ h^−1^ for methanol oxygenates (CH_3_OH and CH_3_OOH) with a remarkable selectivity of 96.5%.^146^ In parallel, Z-scheme architectures have proven equally effective for CH_4_ valorization. A representative example is the W_18_O_49_/g-C_3_N_4_ composite system, in which the formation of a Z-scheme heterojunction was confirmed by XPS analysis, revealing an increased density of oxygen vacancies and enhanced electronic coupling between the two components. Electrochemical characterization, including transient photocurrent measurements and EIS, further corroborated the improved charge separation efficiency. Under visible-light irradiation, the W_18_O_49_/g-C_3_N_4_ composite delivered a CH_3_OH production rate of 62.3 μmol g^−1^ h^−1^, representing a nearly 4-fold enhancement over pristine W_18_O_49_ and a 5-fold enhancement over pristine g-C_3_N_4_.^147^ Together, these examples illustrate the versatility of heterojunction engineering—spanning both S-scheme and Z-scheme architectures—in achieving efficient and selective photocatalytic CH_4_ conversion while offering noble-metal-free, sustainable alternatives for clean energy production and greenhouse gas mitigation.

Despite these encouraging advances, photocatalytic methane conversion remains in its early stages compared with more established reactions such as H_2_ evolution and CO_2_ reduction. Key challenges include the low solubility of CH_4_ in water, which limits mass transfer to the catalyst surface, and the difficulty in achieving high single-pass conversion while maintaining selectivity. Future progress will likely depend on the design of hydrophobic interfacial microenvironments that concentrate CH_4_ near active sites, the development of flow-type photoreactors with enhanced gas-liquid-solid contact, and the integration of *operando* spectroscopic techniques to elucidate the transient evolution of methyl and methoxy intermediates under realistic reaction conditions. From an interfacial engineering perspective, the synergistic combination of S-scheme heterojunctions with atomically dispersed cocatalysts holds promise for achieving the long-standing goal of efficient and selective CH_4_-to-chemicals photoconversion.

### Remediation of emerging recalcitrant contaminants and high-value upcycling of wastes

Historically, research on photocatalytic environmental remediation has predominantly focused on the deep mineralization of conventional pollutants, such as BPA, antibiotics, and organic dyes. For instance, rational facet engineering and the construction of S-scheme heterojunctions have been extensively employed to increase the density of accessible active sites and prolong carrier lifetimes, thereby achieving efficient degradation of these standard targets.^148^ While these pioneering studies have laid a solid foundation for understanding interfacial charge transfer and ROS generation, the traditional “destructive mineralization” strategy falls short of addressing the strategic demands of global sustainability. Currently, aquatic ecosystems are increasingly threatened by complex, recalcitrant emerging contaminants (e.g., plasticizers and fluorinated compounds),^149^^,^^150^ while the exponential accumulation of plastic waste demands urgent intervention.^151^ Consequently, the frontier of interfacial photocatalysis is undergoing a profound paradigm shift: transitioning from the passive elimination of environmental toxicity to the precise cleavage of robust chemical bonds and the constructive upcycling of waste streams.

**Figure 11:**
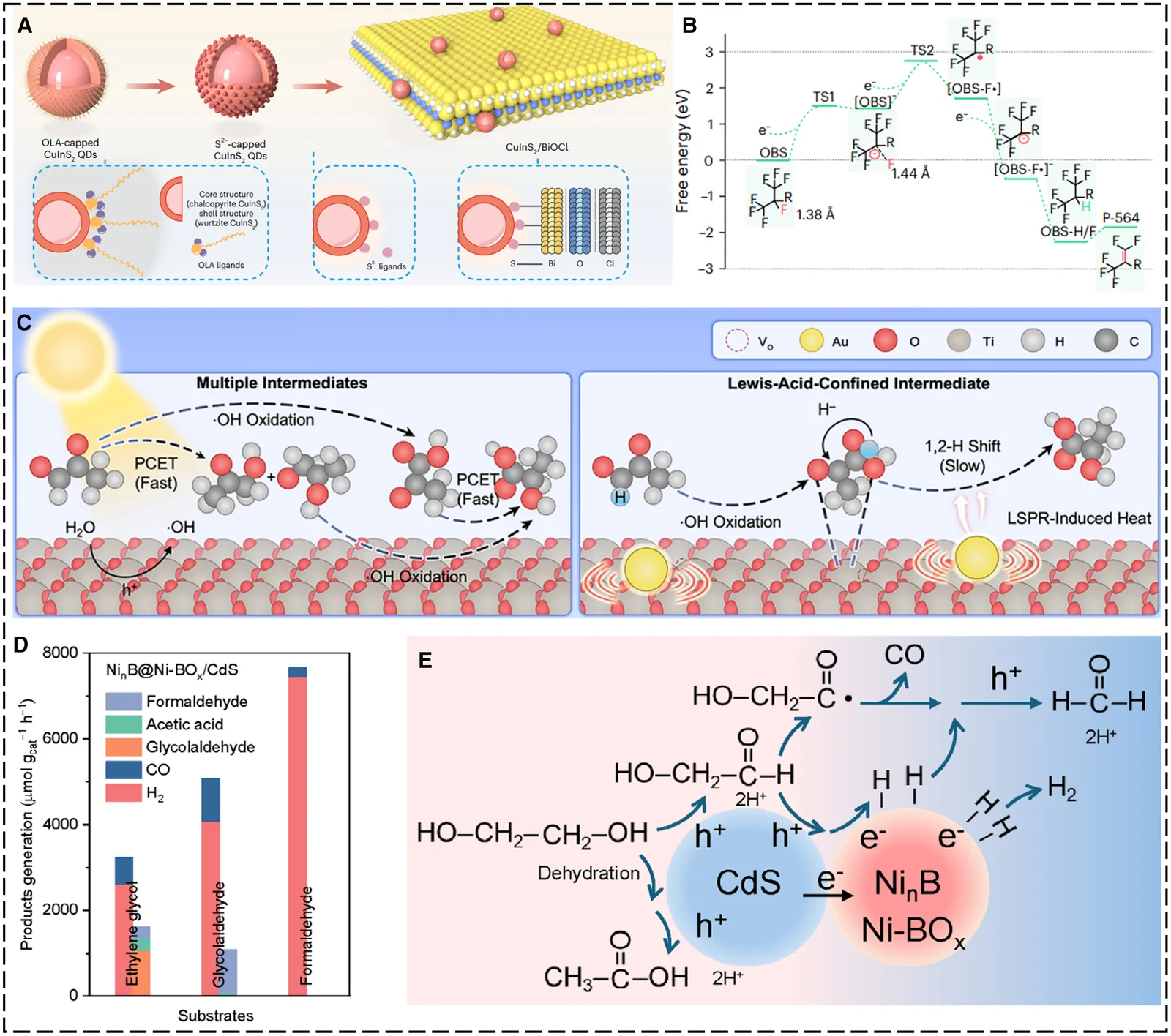
Interfacial photocatalysis for the precise remediation of recalcitrant contaminants and high-value waste upcycling Defluorination of fluorinated pollutants: (A) schematic outlining the fabrication protocol for the CuInS_2_/BiOCl heterojunction, and (B) calculated Gibbs free energy profiles depicting the H/F exchange pathway during OBS defluorination.^154^ Copyright 2026 Springer Nature Ltd. (C) Mechanistic comparison of degradation pathways: conventional photoredox cleavage on pristine TiO_2_ (left) versus the cascade Cannizzaro-type upcycling reaction over the oxygen-vacancy-rich Au/TiO_2_-VO interface (right).^155^ Copyright 2026 American Chemical Society. High-value upcycling of plastics and biomass: (D) product distributions from the photoreforming of diverse feedstocks utilizing the NinB@Ni-BOx/CdS photocatalyst, and (E) proposed reaction pathways for the upcycling of PET-derived ethylene glycol (EG) over the engineered CdS interface.^156^ Copyright 2025 Wiley-VCH GmbH.

In authentic aquatic environments, photocatalysts invariably encounter highly complex microenvironmental interferences. Emerging recalcitrant contaminants, represented by waste plasticizers and per- and polyfluoroalkyl substances (PFAS), pose severe challenges to conventional photocatalytic systems. To achieve the precise eradication of these pollutants in complex matrices (e.g., wastewater rich in dissolved organic matter, DOM), researchers have primarily focused on reconfiguring the interfacial microenvironment. While conventional wisdom dictates that DOM acts as a detrimental hole scavenger, engineering directed hydrogen-bonding networks between the photocatalyst and DOM induces a pronounced steric hindrance. This spatial shielding effectively prevents the undesirable quenching of highly reactive holes and even transforms DOM into an interfacial electron shuttle to synergistically accelerate charge transfer.^152^ Empowered by this microenvironment modulation, researchers developed atomically dispersed catalytic interfaces, such as single-atom cobalt anchored on TiO_2_ (Co_SA_-TiO_2_), to combat waste plasticizers. Boasting near-unity atomic utilization, these single-metal centers achieve the exhaustive degradation of plasticizers within minutes, exhibiting exceptional robust stability across simulated authentic wastewater matrices.^153^ Furthermore, escalating to the even more formidable PFAS (notoriously branded as “forever chemicals”), their extremely inert carbon-fluorine (C-F) bonds (with a dissociation energy of ∼500 kJ/mol) necessitate extreme interfacial band engineering. A recent breakthrough orchestrated a CuInS_2_/BiOCl Z-scheme heterojunction, wherein the robust internal electric field facilitates impeccable spatial charge decoupling (Figure 11A).^154^ This architecture drives a remarkably rare “dual-parallel” degradation mechanism: highly energetic electrons execute electrophilic attacks to initiate defluorination (C-F scission), while isolated holes synchronously trigger the exhaustive truncation of the carbon chain (Figure 11B). This tailored interfacial progression—ranging from hydrogen-bonded microenvironment shielding and precise single-atom activation to Z-scheme-directed robust bond cleavage—provides an illuminating physicochemical paradigm for dismantling the most stubborn environmental contaminants.

If the mineralization of pollutants represents a “defensive battle” in environmental photocatalysis, the upcycling of waste into high-value chemicals represents an “offensive campaign” toward resource circularity. This “Waste-to-Wealth” strategy is purposefully designed to prevent the squandering of intrinsic chemical energy and carbon frameworks. In the realm of biomass valorization, selectively obtaining a single desired product in aqueous media is exceptionally challenging, as intermediates (e.g., pyruvaldehyde) are highly susceptible to over-oxidation or chaotic rearrangements.^157^^,^^158^ To address this, researchers introduced unsaturated titanium Lewis-acid sites, which successfully triggered a cascade reaction coupling photooxidation with a 1,2-hydride shift. This unique interfacial coordination mechanism effectively suppresses detrimental over-oxidation, selectively converting biomass-derived carbohydrates into high-value lactic acid under ambient, neutral conditions (Figure 11C).^155^ Even more disruptive is the upcycling of bulk polyester plastics, such as polyethylene terephthalate (PET). While traditional degradation merely yields low-value mixtures, cutting-edge research has engineered a distinctive core-shell nanoarchitecture by modifying a CdS surface with boron-functionalized nickel species (Ni-B). This interface functions not only as a reductive cocatalyst but also ingeniously mediates an electron-proton cascade transfer. Driven by this synergistic redox mechanism, inert PET plastic waste is successfully photo-reformed into glycolaldehyde—a highly sought-after pharmaceutical intermediate—and syngas (Figures 11D and 11E).^156^ This elegant coupling of environmental remediation with the synthesis of high-value solar fuels and chemicals perfectly embodies the ultimate vision and highest academic caliber of future interfacial photocatalytic material design.

Despite the exhilarating breakthroughs in molecular cleavage—such as the rapid defluorination of PFAS via CuInS_2_/BiOCl Z-scheme heterojunctions or the ultrafast degradation of plasticizers by atomically dispersed Co/TiO_2_—a critical gap remains between these laboratory-scale triumphs and industrial-scale viability. Most current studies predominantly highlight the exceptional intrinsic activity of precisely engineered interfaces in ultrapure water matrices. However, transitioning from the bench to real-world industrial wastewater introduces formidable complexities. In practical scenarios, the presence of complex DOM and high-concentration inorganic salts (e.g., Cl^−^, SO_4_^2−^, and CO_3_^2−^) fundamentally disrupts the interfacial catalytic dynamics. These co-existing species fiercely compete for specific surface active sites and act as potent radical scavengers, drastically quenching the photogenerated ROS and causing a precipitous drop in the reported “minute-level” degradation kinetics. Furthermore, from a techno-economic perspective, the large-scale deployment of these sophisticated photocatalysts remains questionable. The synthesis of single-atom constructs or multi-component Z-scheme heterojunctions often demands expensive precursors, harsh conditions, and intricate procedures with low yields. Compared to mature, easily scalable traditional advanced oxidation processes (AOPs) such as Fenton or ozonation, the economic competitiveness of these highly engineered nano-interfaces is currently limited. Therefore, future interfacial engineering must urgently pivot from a purely “activity-driven” paradigm to a “robustness-driven” approach. Developing anti-fouling, selectively adsorptive interfaces capable of maintaining long-term structural integrity in realistic, highly saline matrices represents the ultimate touchstone for the commercialization of environmental photocatalysis.

## Conclusions

The rational design of interfacial catalytic materials has demonstrably transcended the limitations of traditional semiconductors, establishing a robust physicochemical framework for high-efficiency solar energy utilization. As highlighted, the strategic regulation of interfaces—ranging from macroscopic heterojunction alignments to atomic-level coordination environments—addresses both the thermodynamic and kinetic bottlenecks of photocatalysis. The implementation of S-scheme architectures elegantly resolves the long-standing paradox between charge separation efficiency and redox potential preservation. Furthermore, the introduction of asymmetric single-atom dopants, non-metal active centers, and spatially decoupled cocatalysts acts as highly specific “catalytic pockets”, precisely steering complex PCET pathways. Driven by the synergistic combination of advanced *operando* characterizations (e.g., fs-TAS, ISI-XPS, and *in situ* FTIR) and DFT computations, the once-opaque “black-box” of photocatalysis has been vividly decoded, accelerating the transition from empirical trial-and-error to targeted, mechanistic-driven design. Empowered by these interfaces, remarkable breakthroughs have been achieved in critical applications, evolving from basic hydrogen evolution and organic dye degradation to the selective C-C coupling in CO_2_ reduction, the targeted C-F bond cleavage of PFAS, and the dual-functional upcycling of waste plastics and biomass.

In conclusion, interfacial photocatalysis has matured into a sophisticated, highly interdisciplinary science. By bridging the critical gaps between precise atomic synthesis, *operando* kinetic tracking, and macroscopic chemical engineering, the rational design of interface photocatalysts will hopefully serve as a cornerstone in the impending transition toward a sustainable, carbon-neutral, and pollution-free global future.

## Acknowledgments

This work was supported by the National High Technology Research and Development Program of China (2021YFF1200200) and start-up funds from Tianjin University, China.

## Author contributions

Conceptualization, data curation and writing - original draft: C.C. and W.Q. Data curation and figure design: C.H. Conceptualization, data curation, writing - editing, and supervision: G.G. Writing - review and editing, funding acquisition, and supervision: H.M. Y.

## Declaration of interests

The authors declare no competing interests.
